# Interactions between the mRNA and Rps3/uS3 at the entry tunnel of the ribosomal small subunit are important for no-go decay

**DOI:** 10.1371/journal.pgen.1007818

**Published:** 2018-11-26

**Authors:** Carrie L. Simms, Kyusik Q. Kim, Liewei L. Yan, Jessica Qiu, Hani S. Zaher

**Affiliations:** Department of Biology, Washington University in St. Louis, St. Louis, Missouri, United States of America; University of California, San Francisco, UNITED STATES

## Abstract

No-go Decay (NGD) is a process that has evolved to deal with stalled ribosomes resulting from structural blocks or aberrant mRNAs. The process is distinguished by an endonucleolytic cleavage prior to degradation of the transcript. While many of the details of the pathway have been described, the identity of the endonuclease remains unknown. Here we identify residues of the small subunit ribosomal protein Rps3 that are important for NGD by affecting the cleavage reaction. Mutation of residues within the ribosomal entry tunnel that contact the incoming mRNA leads to significantly reduced accumulation of cleavage products, independent of the type of stall sequence, and renders cells sensitive to damaging agents thought to trigger NGD. These phenotypes are distinct from those seen in combination with other NGD factors, suggesting a separate role for Rps3 in NGD. Conversely, ribosomal proteins ubiquitination is not affected by *rps3* mutations, indicating that upstream ribosome quality control (RQC) events are not dependent on these residues. Together, these results suggest that Rps3 is important for quality control on the ribosome and strongly supports the notion that the ribosome itself plays a central role in the endonucleolytic cleavage reaction during NGD.

## Author summary

In all organisms, optimum cellular fitness depends on the ability of cells to recognize and degrade aberrant molecules. Messenger RNA is subject to alterations and, as a result, often presents roadblocks for the translating ribosomes. It is not surprising, then, that organisms evolved pathways to resolve these valuable stuck ribosomes. In eukaryotes, this process is called no-go decay (NGD) because it is coupled with decay of mRNAs that are associated with ribosomes that do not ‘go’. This decay process initiates with cleavage of the mRNA near the stall site, but some important details about this reaction are lacking. Here, we show that the ribosome itself is very central to the cleavage reaction. In particular, we identified a pair of residues of a ribosomal protein to be important for cleavage efficiency. These observations are consistent with prior structural studies showing that the residues make intimate contacts with the incoming mRNA in the entry tunnel. Altogether our data provide important clues about this quality-control pathway and suggest that the endonuclease not only recognizes stalled ribosomes but may have coevolved with the translation machinery to take advantage of certain residues of the ribosome to fulfill its function.

## Introduction

The elongation phase of translation is an imperfect process, during which the ribosome moves with irregular speed along the mRNA template [1]. By and large the elongation speed is determined by sequence and structural features of the coding sequence. For instance, the identity of the A-site codon is known to have a drastic effect on the rate of protein synthesis depending on the availability of its partner tRNA and the nature of the codon-anticodon base-pairing interaction [2, 3]. Furthermore, the chemical characteristics of the locally-encoded amino acids have been shown to regulate the rate of protein synthesis based on the manner they interact with the exit tunnel of the ribosome [4]. mRNAs are also known to harbor local secondary structures that can slow down the ribosome as it unwinds them [5, 6]. Regardless of the underlying mechanism, the fluctuating rate of protein synthesis along an mRNA molecule appears to serve important biological functions such as promoting appropriate co-translational protein folding and ensuring that the encoded protein is targeted to the correct destination in the cell [7–12].

In contrast to this “programmed” regulation of ribosome traffic, the ribosome often encounters unwanted obstacles that severely hinder its progression and in some cases stall protein synthesis all together [13, 14]. Most of these impediments are typically associated with defects in the mRNA, including stable secondary structures, stretches of rare and inhibitory codons, as well as truncations and chemical damage [3, 15–17], [18]. Because multiple ribosomes are typically translating a single mRNA at any given point, one stalled ribosome is likely to impede the progression of multiple upstream ribosomes. As a result, if left unresolved, these stalling events have the potential to severely reduce cellular fitness. Notably, the stalling of the ribosome itself is not such a detriment to the cell as is the loss of valuable ribosomes from the translating net pool [13, 14]. In eukaryotes, the evolutionary solution to this predicament was No-Go Decay (NGD) [15] as a means to dissociate stalled ribosomes [19–21]. It is thought that over time, this mechanism was expanded on to include mRNA surveillance to dispose of the aberrant mRNA. In particular, the mRNA undergoes an endonucleolytic cleavage upstream of the stall site. The resulting deadenylated 5’-end and uncapped 3’-end pieces are then rapidly degraded by the exosome and Xrn1, respectively [3, 15–17].

Initial studies on NGD in yeast focused on the two factors Dom34 (Pelota in mammals) and Hbs1 [15, 22, 23]. These factors are homologs of the termination factors eRF1 and eRF3, respectively. Early reports of NGD hinted at a role for the factors in mediating the endonucleolytic cleavage of the mRNA near the stalled ribosome [15, 24]. However, later studies by the same group and others showed the cleavage to take place in the absence of the factors [22] leaving the question of the role of the factors in the process unanswered. Interestingly prior to the discovery of NGD, genetics studies suggested that Dom34 and Hbs1 are important in maintaining ribosome homeostasis of the cell [25]. To this end, both factors become essential or near-essential when ribosomes are depleted either by knocking down certain ribosomal proteins or under conditions when ribosomes are sequestered [25–27]. These observations are consistent with biochemical studies using a yeast translation reconstituted system, which showed the factors to be responsible for dissociating ribosomes into their respective subunits [18]. This splitting activity of Dom34-Hbs1 was also found to be much more efficient in the presence of Rli1 (ABCE1 in mammals) [20, 21]. *In vivo* data also supported this model for the role of the three factors in dissociating ribosomes [16]. Hence, this rescuing/recycling activity of these factors rationalizes the effect of their deletion on ribosome availability, especially under stress conditions.

In addition to ribosome rescue and degradation of the aberrant RNA, NGD is closely linked to a newly discovered protein-quality-control process termed ribosome quality control (RQC). This process is responsible for degrading the incomplete nascent protein resulting from stalled translation [28–34]. RQC proceeds after the splitting action of Dom34-Hbs1-Rli1, which results in a peptidyl-tRNA-associated large-ribosome subunit. This atypical form of the 60S subunit is recognized by the E3 ligase Ltn1 (Listerin in mammals) alongside Rqc2 (formerly Tae2) [30, 33, 35]. Ltn1 ligates ubiquitin chains to the nascent peptide as it is attached to the tRNA on the large subunit. The ubiquitinated nascent peptide is then extracted and delivered to the proteasome for degradation through the action of Rqc2 and Cdc48 (and its adaptor proteins Ufd1 and Npl4). Two additional factors, the ribosome-associated Asc1 and the E3 ligase Hel2 (Rack1 and Znf598 in mammals, respectively), also appear to be important for proper RQC function. Both factors are important for ribosomal protein ubiquitination and appear to play a role during stalling [36, 37]. In particular, deletion of either factor results in increased readthrough of stall sequences [38, 39]. How regulatory ribosomal protein ubiquitination interconnects with RQC and NGD is currently poorly understood.

Even though the consequences of ribosome stalling in eukaryotes was initially described in the context of its impact on mRNA steady state levels [15], as detailed above we know far more about its entanglement with ribosome rescue and quality control of the associated nascent peptide. More specifically, degradation of the mRNA is initiated by endonucleolytic cleavage, but the identity of the endonuclease remains elusive. This in turn has precluded further critical mechanistic dissections of NGD. Some of these outstanding important questions are: 1) How does the endonuclease recognize stalled ribosomes? 2) Is it associated with the ribosome? 3) Does it have a specificity for certain mRNAs 4) How is its function activated? 5) Can NGD be used to regulate gene expression? Work from our group recently provided some clues about the cleavage reaction. Using reporters and genetic manipulation of yeast we showed that the physical act of ribosome collision is important for initiating the process of RNA degradation and ribosome rescue during no-go decay (NGD) [40]. High-resolution mapping of the cleavage products also provided some important clues about the potential role of the ribosome in the reaction. Namely, cleavage appears to take place well upstream of the lead stalled ribosome with the closest most prominent one being ~45 nt upstream of the stall site. As ribosomes are likely to be stacked on the mRNA, this suggested the possibility that the cleavage is taking place inside the ribosome [18, 41]. Multiple regions of the ribosome make intimate contact with the mRNA. Most noteworthy among these is the mRNA entry tunnel, which encompasses residues of the ribosomal proteins Rps3/uS3 and Rps2/uS5 [42]. In eukaryotes additional contacts are made by helices 18 and 14 of the 18S rRNA, whereas in bacteria these contacts are carried out by Rps4/uS4 (orthologous to Rps9 in yeast and humans) [42–44]. In the entry tunnel, Rps3’s contacts with the mRNA stand out because they appear to be almost universally conserved and form an integral part of the helicase domain of the ribosome [42]. Furthermore, the protein has been implicated in translation initiation during the rearrangement of the small subunit that allows for the opening of the ribosomal mRNA binding channel and subsequent scanning of the mRNA [45] as well as start-codon selection [46].

Here we show the entry tunnel of the ribosome to play an important role during NGD. Mutation of the residues of *RPS3* that form part of the entry tunnel, which have also been implicated in the helicase activity of the ribosome, were found to significantly reduce the accumulation of cleavage products. This effect on cleavage efficiency to a large extent was independent of the identity of the stall site. Combining these mutations with factors involved in other aspects of NGD revealed that the entry tunnel is also likely to be important in ribosome rescue. Our findings provide new insights into how quality control mechanisms evolved to integrate into fundamental biological machines.

## Results

### Mutation of the entry-tunnel residues of *RPS3* affects cleavage of NGD reporters

**Fig 1:**
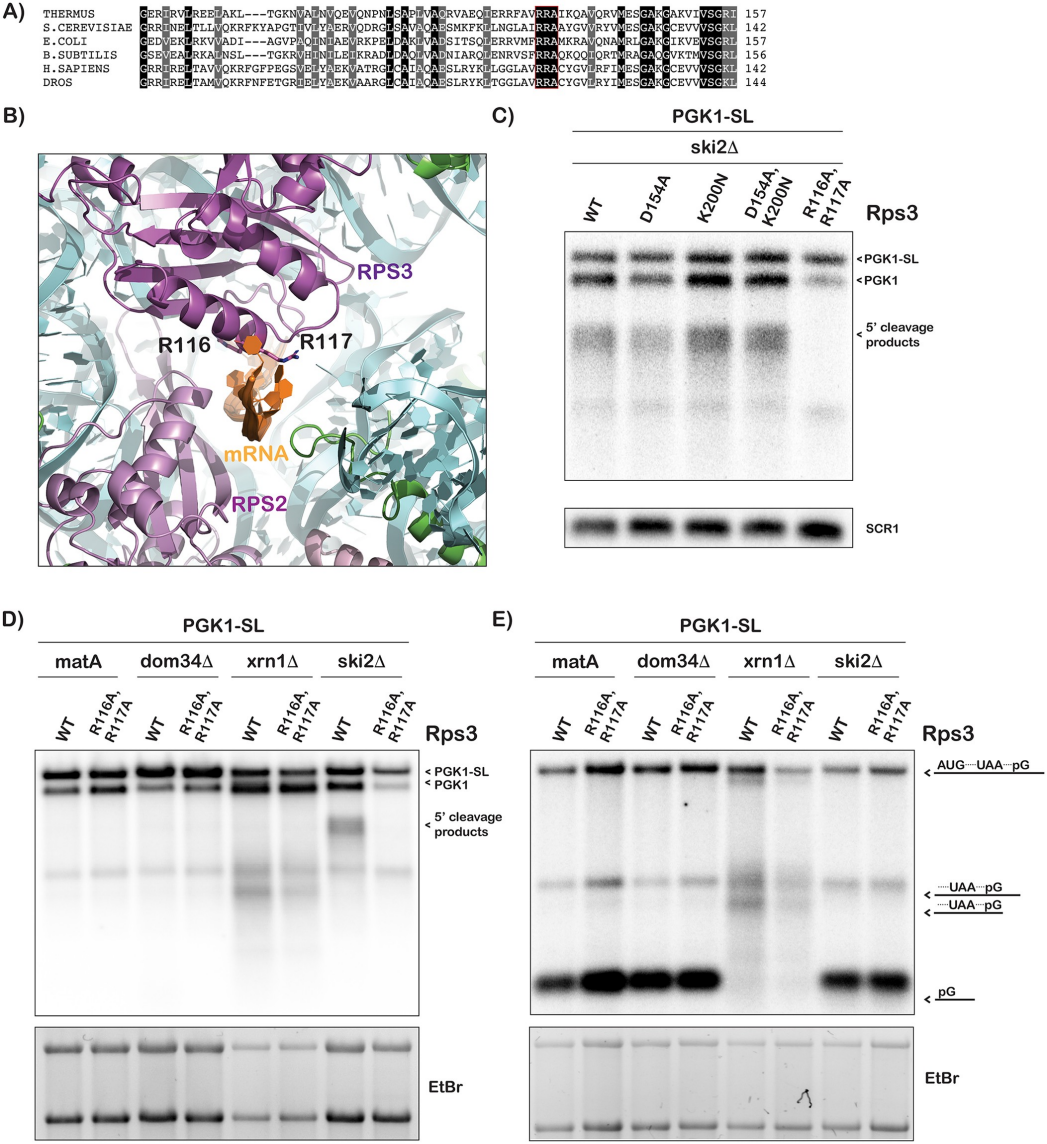
Conserved residues in *RPS3* that affect the endonucleolytic cleavage reaction. (**A**) Alignment of partial Rps3 sequences from bacteria species, yeast, Drosophila, and human, with conserved residues highlighted including yeast R116 and R117, shown boxed in red. (**B**) Structure of the entry tunnel of the ribosome, with position of Rps3 residues R116 and R117 shown (PDB ID 5AJ0). (**C**) Northern analysis of 5’ fragments derived from a PGK1-stem loop (SL) reporter in *ski2Δ* strains harboring mutations in *RPS3*. Accumulation of 5’ cleavage fragments is significantly reduced in the R116A/R117A mutant. (**D**) Northern analysis of 5’ fragments accumulation from PGK1-SL in the indicated strains with either wild type or mutant *RPS3*. Corresponding ethidium-bromide stained agarose gel (bottom panel). (**E**) Northern analysis of 3’ fragments accumulation from PGK-SL in the indicated strains with wild type or mutant *RPS3*. The fragments are labeled as in (Chen et al. 2010). Corresponding ethidium-bromide stained agarose gel (bottom panel).

To address a potential role for Rps3 in the cleavage reaction, we introduced a number of mutations to the protein and assessed their effect on cleavage of stalling reporters. Our choice of residues for the mutations was motivated by three criteria: they had to be conserved, made intimate contacts with the mRNA and have basic or acidic side chains (Fig 1A and 1B). This led us to Arg116 (R116) and Arg117 (R117). In addition to these, we also analyzed two residues that have been suggested to be important for Rps3’s extra-ribosomal activity in DNA repair [47–51], Asp154 (D154) and Lys200 (K200). Mutation of these residues abolishes the 8-oxoguanosine glycoslase and AP/endonuclease activities of the protein [51]. The variant-yeast strains were generated by introducing mutations to the chromosomal copy of *RPS3* (see Methods) in different backgrounds of deletions and mutations. All in all, we generated the following mutants: Arg116 and Arg117 were substituted by Ala residues (R116A/R117A), Asp154 was substituted by an Ala residue (D154A), Lys200 was substituted by an Asn residue (K200N) and finally we generated a double mutant D154A/K200N. Of these the R116A/R117A mutation was notable as the side chain of these residues are projected into the entry tunnel of the ribosome and make electrostatic interactions with the mRNA (Fig 1B).

Next, we assessed the effect of these mutations on the cleavage of NGD substrates. We initially used an NGD reporter, which harbors a stable stem loop in the *PGK1* coding sequence and was originally designed by Parker and colleagues. The stem loop presents a robust obstacle for the ribosome and is subject to an endonucleolytic cleavage as evidenced by the accumulation of 5’ and 3’ fragments when the exosome and Xrn1 are inactivated, respectively [15]. Indeed, similar to what was observed by us and others [15, 16, 40], in the *ski2Δ* strain- which is defective for 3’-5’ mRNA degradation- northern analysis of cells expressing PGK1-SL revealed substantial accumulation of 5’-fragments (Fig 1C). The D154A and K200N mutations in *RPS3*, which have been suggested to be important for an AP endonuclease activity [51], had no observable effect on the cleavage efficiency and appear to play no role in NGD. In contrast, the R116A/R117A mutations appear to reduce the accumulation of cleavage fragments and also increased heterogeneity among these products (Fig 1C). Interestingly, the mutations also appear to affect the steady-state levels of endogenous PGK1 transcript (Fig 1C). Regardless, these observations suggest that residues of Rps3 that interact with the mRNA in the entry tunnel are important during NGD.

The effects of the R116A/R117A mutations on the cleavage reaction were further studied in the context of other deletions that alter different aspects of NGD. Namely, we introduced these mutations into *dom34Δ* and *xrn1Δ* strains in addition to the wild-type parent strain. As expected, expression of the PGK1-SL in these strains does not result in the accumulation of 5’-fragments and the R116A/R117A mutations have no effect. As a control, these fragments were seen in the *ski2Δ* background and the *rps3* mutations significantly reduced their levels (Fig 1D). Production of the 3’-fragments, as expected, was seen in the absence of *XRN1* and their levels diminished in the presence of the *RPS3* mutations, albeit to a lower extent than that seen for the 5’ fragments (Fig 1E). These latter observations suggested that the R116A/R117A mutations do not completely inhibit cleavage and that they may affect other aspects of NGD.

### The R116A/R117A mutations affect the stability of an NGD reporter mRNA

**Fig 2:**
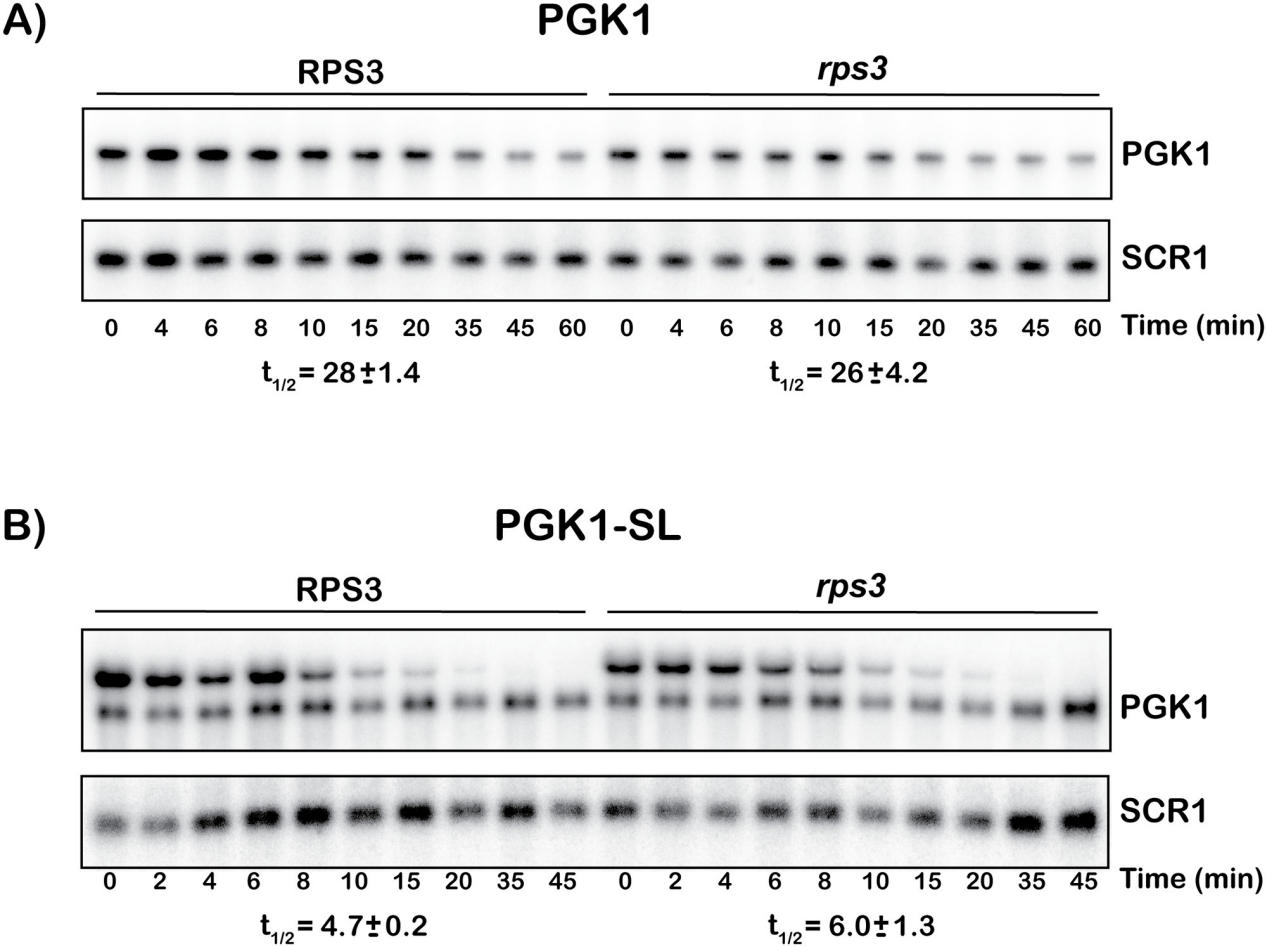
Mutations in *RPS3* stabilize a PGK1-SL NGD reporter. (**A**) Northern analysis of time course measuring decay of a control PGK1 reporter (top panel) with blot re-probed for control RNA *SCR1* (bottom panel). No substantial difference in half-lives was observed between wild type and mutant *RPS3*. (**B**). In the presence of R116A/R117A mutations, the turnover of the PGK-SL stem loop reporter is reduced by about one third. Half-lives reported below each panel are an average ± SD.

To provide further support for a role for the entry tunnel residues of Rps3 during NGD, we next examined the effect of the mutations on the stability of the PGK1-SL mRNA. Our reporters are expressed under the control of the *GAL1* promoter, and as a result transcriptional-shutoff by shifting cells to glucose-containing media was used to measure the decay rate of the reporter mRNAs. As a control, we initially measured the decay rate of a non-NGD reporter (PGK1), which does not harbor any stalling sequence. The mutations were found to have little effect on the decay rate of the PGK1 mRNA reporter (Fig 2A); we measured half-lives of 28 ± 1.9 and 26 ± 4.4 minutes in the WT and the *RPS3*-mutant stains, respectively. As expected, the PGK1-SL mRNA decays with a faster rate relative to its PGK1 parent (Fig 2B). Its half-life of 4.7 ± 0.2 minutes is similar to previously published reports [15]. Here the *RPS3* mutations result in a moderate but reproducible increase in reporter half-life to 6.0 ± 1.3 minutes, suggesting greater stabilization of the PGK1-SL mRNA (Fig 2B). Hence, these findings add additional support for the entry tunnel of the ribosome playing a role in mRNA-surveillance during NGD, whereby loss of interactions with the mRNA leads to stabilization of mRNAs harboring stalls.

### The effect of R116A/R117A mutations on the cleavage reaction is independent of the stalling sequence

**Fig 3:**
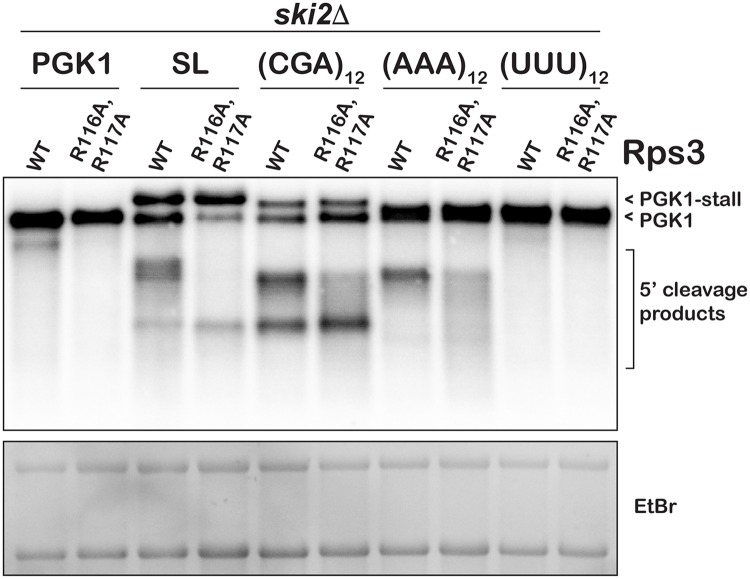
Mutations in *RPS3* affect cleavage efficiency independent of stall sequence. Northern analysis of 5’ fragments accumulation from *ski2Δ* strains, with and without mutations in *RPS3*, carrying different stalling reporters. PGK1 contains a PGK1 mRNA without any additional sequence; SL contains a stem loop at position 1040 of PGK1; (CGA)_12_, (AAA)_12_, and (UUU)_12_ indicate the corresponding codons were inserted at position 950 of PGK1. Corresponding ethidium-bromide stained agarose gel is shown (bottom panel).

So far, our analysis has focused on one type of stall—a stable RNA secondary structure in the form of a stem loop. Since the mutations under investigation here are important for the helicase function of the ribosome, any effect we saw on the cleavage reaction could be explained by defects in the unwinding activity of the ribosome and not in NGD. To rule out this potential explanation, we used two other reporters that had 12 stretches of the inhibitory arginine CGA or lysine AAA codons. Both are known to efficiently block translation and are not predicted to form secondary structures [3, 15, 23]. These new reporters were introduced to wild-type or mutant *RPS3* yeast strains in the *ski2Δ* background. As expected, the CGA and AAA reporters accumulated 5’-fragments in the wild-type *RPS3* strain, whereas the control UUU reporter did not (Fig 3). Similar to what we observed for the SL reporter, the R116A/R117A mutations significantly reduced the 5’-fragments levels for the CGA and AAA reporters, suggesting that the entry tunnel residues affect the accumulation of cleavage fragments independent of the type of stall (Fig 3). Interestingly, however, unlike the SL reporter, for which we observe an almost complete loss of cleavage products when *RPS3* was mutated, cleavage fragments resulting from the CGA and AAA reporters were still visible but instead were heterogeneous in nature (Fig 3). This also made it difficult to perform any meaningful quantification. This is likely due to cleavage fragments produced by inefficient initial cleavage reactions, which lead to ribosome queuing upstream of the lead stalled ribosome.

Ski7, a component of the exosome in yeast, has been implicated in non-stop decay (NSD) [52–54]; given the similarities between NSD and NGD, the mutations in *RPS3* could potentially affect the function of Ski7. To address this possibility, we deleted *SKI7* from the wild-type, *dom34Δ* and *ski2Δ* stains in the absence and presence of the *RPS3* mutations and assessed its effect on NGD efficiency from the SL reporter. We observed no significant changes to the accumulation of the 5’-fragments due to the *SKI7* deletion suggesting that the entry tunnel residues do not affect the function of the factor (S1 Fig).

As mentioned earlier, in addition to Rps3, the mRNA entry tunnel of the small subunit also encompasses conserved residues of the ribosomal protein Rps2 [42, 55]. Namely the side-chain of Glu120 of the yeast protein protrudes into the entry tunnel and is likely to interact with the mRNA downstream of the A site (S1 Fig). Consequently, we determined whether this residue contributes to NGD or not. We mutated Glu120 to Ala in the *ski2Δ* strain and evaluated its effect on NGD cleavage efficiency. In contrast to the *RPS3* mutations, the *RPS2* mutation had no noticeable effect on the cleavage reaction; we observed comparable levels of 5’-fragments accumulation from the SL reporters in the *RPS2* wild-type and mutant strains (S1 Fig). It thus appears that the changes to NGD we observe in the presence of the *RPS3* mutations are the result of Rps3-dependent effects, and likely not from general alterations to the mRNA-entry tunnel.

### Dom34 and Asc1 modify the effects of the R116A/R117A mutation

**Fig 4:**
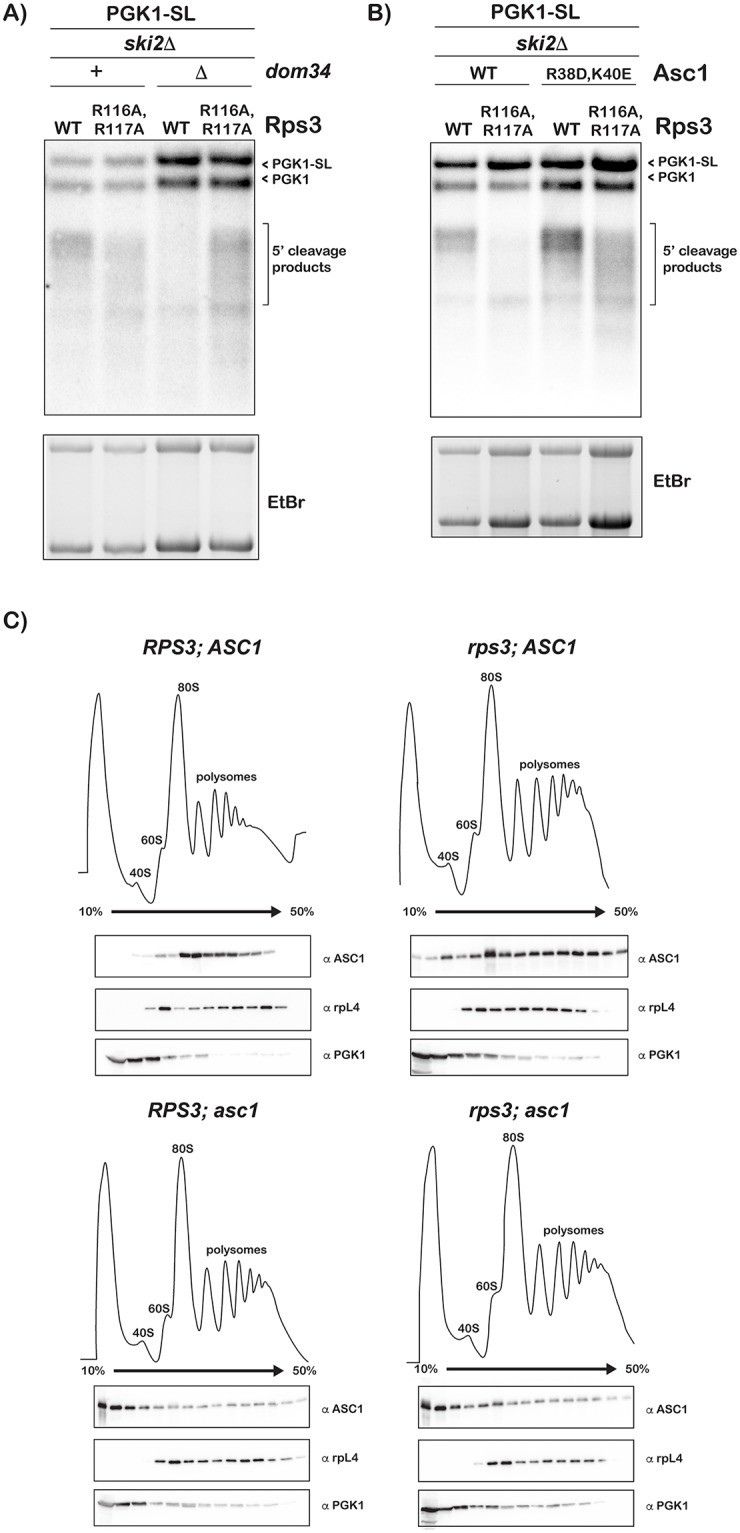
Assessing the effect of Dom34 and Asc1 modification on the cleavage reactions in the presence of the *RPS3* mutations. (**A**) Northern analysis of 5’ fragments produced from the PGK1-SL reporter in cells with *DOM34* deleted and *rps3* R116A/R117A mutations. Cleavage efficiency is restored in the *dom34Δ*; *rps3* R116A, R117A cells relative to the *dom34Δ* alone cells, however the products appear smeared compared to those from the *rps3* mutant cells, indicating increased heterogeneous cleavage. (**B**) Northern analysis of 5’ fragments generated by cells with the *asc1* R38D/K40E and *rps3* R116A/R117A mutations. Mutations in *asc1* restore cleavage in a *rps3* mutant strain, but also lead to greater heterogeneous cleavage. In both A and B, bottom panels show the corresponding ethidium-bromide stained agarose gel. (**C**) Polysome profiles of cells from the indicated strains. Shown below in each panel are western analyses across the polysome fractions of: Asc1 (top panels), Rpl4 (middle panels) and Pgk1 (bottom panels). As expected the wild type Asc1 protein’s interaction with the ribosome is not affected by the *RPS3* mutations (top two panels). As expected, Asc1 R38D/K40E resides predominantly in the light fractions, unbound by the ribosome (bottom two panels). Rpl4 and Pgk1 are included as controls and remain unchanged in the presence of the *rps3* mutations. Blots shown represent three biological replicate experiments.

Initial reports of NGD suggested that Dom34 plays a role in the cleavage reaction due to the loss of the cleavage products accumulation when the factor is deleted [15, 24]. Later studies, however, showed that the protein together with Hbs1 and ABCE1 dissociates stalled ribosomes [19]. In its absence ribosomes pile up on the mRNA leading to multiple cleavage events upstream of the lead stalled ribosome, which run as a long smear on a gel that appears to result in loss of cleavage efficiency [16]. Furthermore, overexpression of certain ribosomal proteins restored cleavage in the absence of *DOM34*, suggesting that the protein is involved in maintaining ribosome homeostasis [22]. To gain further insights into the role of the entry-tunnel residues in ribosome rescue, we deleted *DOM34* from our *RPS3*-mutant strains and assessed its effect on the accumulation of 5’-fragments from the PGK1-SL reporter. As had been seen by others, deletion of *DOM34* appeared to result in a loss of cleavage [16]. Interestingly the same deletion in the presence of the R116A/R117A mutations appears to restore cleavage with one caveat; the fragments are much more heterogeneous relative to those observed under normal conditions (Fig 4A). In particular, the products were observed to form a long smear on agarose gels. It seems that, under conditions where ribosome rescue is inhibited, mutation of the entry tunnel residues leads to a spreading of cleavage events well upstream of the stall site.

To provide further support for this notion, we examined the effect of mutations in *ASC1* on cleavage in conjunction with the *RPS3* mutations. Asc1 is a ribosome-associated protein that has been implicated in multiple aspects of ribosome quality control processes including NGD [38, 56–58]. For instance, cryoEM structures of a Dom34-Hbs1-bound ribosome revealed the factor to interact with Dom34 suggesting that it is critical for NGD [59, 60]. In addition, recent data from the Inada group showed that the factor is important for sequential endonucleolytic cleavage during non-stop decay (NSD) in the absence of *DOM34* [58]. Instead of deleting *ASC1*- which harbors a snoRNA gene in its intron- from our *rps3* strains, we opted to introduce the R38D/K40E mutations into the chromosomal copy of the gene. These mutations are known to affect the association of the factor with the ribosome and phenocopy its deletion in NGD [61]. Similar to the effect we saw in the *dom34Δ* background, the *ASC1* mutations resulted in the accumulation of heterogeneous 5’-fragments from the PGK1-SL NGD substrate in the presence of the R116A/R117A mutations (Fig 4B). To verify that the effect on NGD we observe with the *RPS3* mutants are not due to decreased association of Asc1 with the ribosome, we carried out polysome analysis and used western analysis to look at the binding of Asc1 to ribosomes. As can be seen in Fig 4C, ribosomal occupancy by wild-type Asc1 is not significantly altered by the mutations in *RPS3*; similar to the wild-type, the protein was found to primarily associate with the polysomes in the presence of the *RPS3* mutation (top panels). As a control, the R38D/K40E mutant was observed in the light fractions of the sucrose gradient, that is not ribosome-associated, regardless of *RPS3* status (bottom panels). We should note, though, Asc1 participates in a multitude of processes on the ribosome including translation of short ORFs, stall clearance and ribosomal protein ubiquitination [37, 38, 56–58, 62]. As a result, any interpretation of its consequence on NGD is likely to be complicated by the larger context of its effect on ribosome function.

How inhibition of ribosome rescue either by deletion of *DOM34* or mutation of *ASC1* restores cleavage efficiency to entry-tunnel mutants, albeit with a distinct signature of heterogeneous product accumulation, is difficult to interpret. One plausible explanation is that the R116A/R117A mutations inhibit the accumulation of cleavage fragments and under normal conditions ribosome rescue is fast enough to dissociate stalled ribosomes, which results in the observed disappearance of cleavage products. When rescue slows down due to reduced cleavage kinetics, ribosomes accumulate on the mRNA, initiating cleavage further upstream of the stall sequence.

### High-throughput sequencing of the 5’-fragments reveals spreading of cleavage events in the presence of the *RPS3* mutations

**Fig 5:**
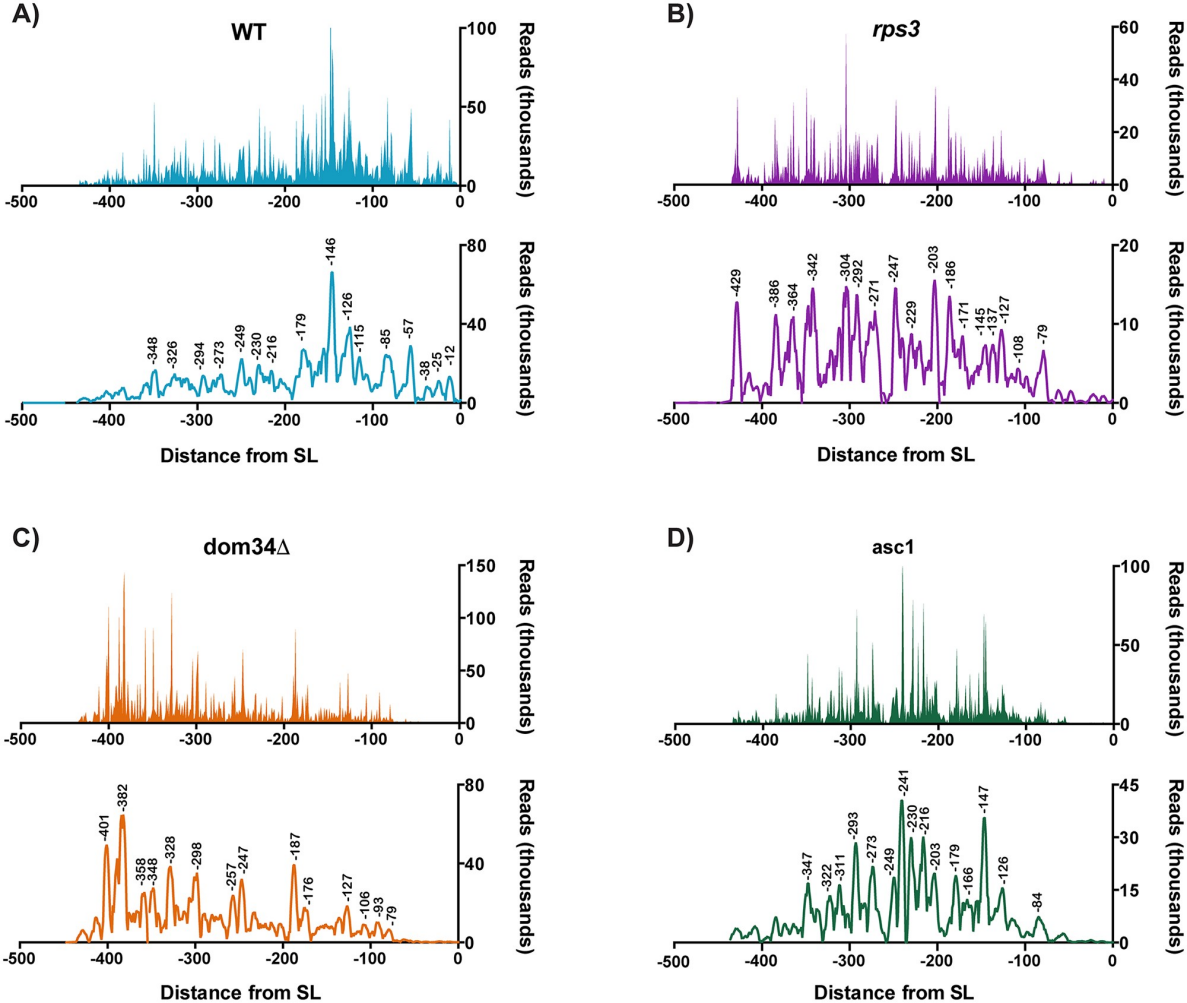
Large scale sequencing reveals changes in cleavage patterns in the presence of *RPS3* mutations. Plot of sequencing reads of 3’ RACE products from the indicated strains, each expressing PGK1-SL. All strains are in a *ski2Δ* background. Each point represents a single read, mapped relative to the stall site and the bottom plot in each panel denotes smoothed data, produced using a 5-point quadratic polynomial. Peaks with values at the 75^th^ quartile or above are labelled as position relative to the stall sequence. (**A-B**) Compared to wild type (**A**), *RPS3* mutations (**B**) result in highly heterogeneous cleavage without a predominant peak. (**C-D**) Deletion of *DOM34* (**C**) or mutations in *ASC1* (**D**) each produce greater heterogeneity of cleavage products with distinct patterns compared to each other and to wild type. Data in panel A is adapted from Simms et al [62].

Our Northern analysis of the NGD-cleavage products suggested that the R116A/R117A mutations affect cleavage fragments accumulation and result in ribosome queueing upstream of the stall site. This pile-up of ribosomes, in turn, results in cleavage reactions even farther upstream leading to diffusion of the NGD intermediates. We provided further support for these ideas by conducting high-throughput sequencing to map the 3’-end of the 5’-NGD fragments. Briefly, total RNA was isolated from strains harboring either the *RPS3* mutants, *dom34Δ*, or *ASC1* mutants in the *ski2Δ* background, each expressing one of the three NGD reporters- SL, (CGA)_12_ and (AAA)_12_. An adenylated DNA oligonucleotide was ligated to the 3’-end of the RNA samples, which was used to prime reverse transcription. The resulting cDNA was then amplified using a PGK1-specific 5’-primer and subjected to high-throughput sequencing using the Illumina Hiseq 2500 platform (GEO accession: GSE117652). Similar to what we have reported earlier [40], for otherwise wild-type cells, the 5’-fragments resulting from the PGK1-SL reporter mapped well upstream of the stall in all strains regardless of the mutational background (Fig 5). However, mapping of the fragments from the R116A/R117A mutant cells revealed extensive spreading of the cleavage events (Fig 5B). More specifically, whereas in the wild-type *RPS3* background we observe one predominant peak near the ~150-nt upstream mark, in the *rps3* mutant background, no predominant peak was observed (Fig 5B). Instead, fragments mapped throughout a 500-nt region upstream of the stall site and multiple peaks were observed with a near 30-nt periodicity. Interestingly, in the *dom34Δ* and the *asc1* cells, the fragments displayed distinct mapping patterns relative to the wild-type and *rps3* cells as well as to each other. Similar to what was observed for the *rps3* mutant cells, in the *dom34Δ* cells the predominant peak at ~150-nt is lost, but here the distance between the peaks increased to 40–60 nt (Fig 5C). This is consistent with the role of Dom34 in rescuing ribosomes that run to the end of the transcript following endonucleolytic cleavage on NGD reporters. Since multiple ribosomes appear to be required for efficient cleavage, the reaction would be expected to occur every ~45-nt- with the lead ribosome protecting 15-nt, while the one behind protects 30-nt. In clear distinction to both the *rps3* and the *dom34Δ* cells, mapping of the 5’-fragments from the SL reporter was not as diffuse in the *asc1* mutant cells. Instead, only one additional predominant peak (relative to the wild-type cells) was observed at ~250 nt upstream (Fig 5D). Differences in cleavage patterns from the WT, *rps3* and *dom34Δ* cells were also evident for 5’-fragments obtained from the (CGA)_12_ reporter, and to a lesser extent for (AAA)_12_ reporter (S2 Fig). We note that for both the (CGA)_12_ and (AAA)_12_ reporters, fewer reads were mapped in the *rps3* cells, presumably due to decreased cleavage efficiency. These differences between the R116A/R117A mutant, and the *DOM34* and *ASC1* mutants suggest that the entry tunnel of Rps3 affects different aspects of NGD relative to these factors. It is also consistent with our model that these residues are important for the endonuclease function.

### Polysome analysis reveals that the *RPS3* mutations do not affect ribosome homeostasis

**Fig 6:**
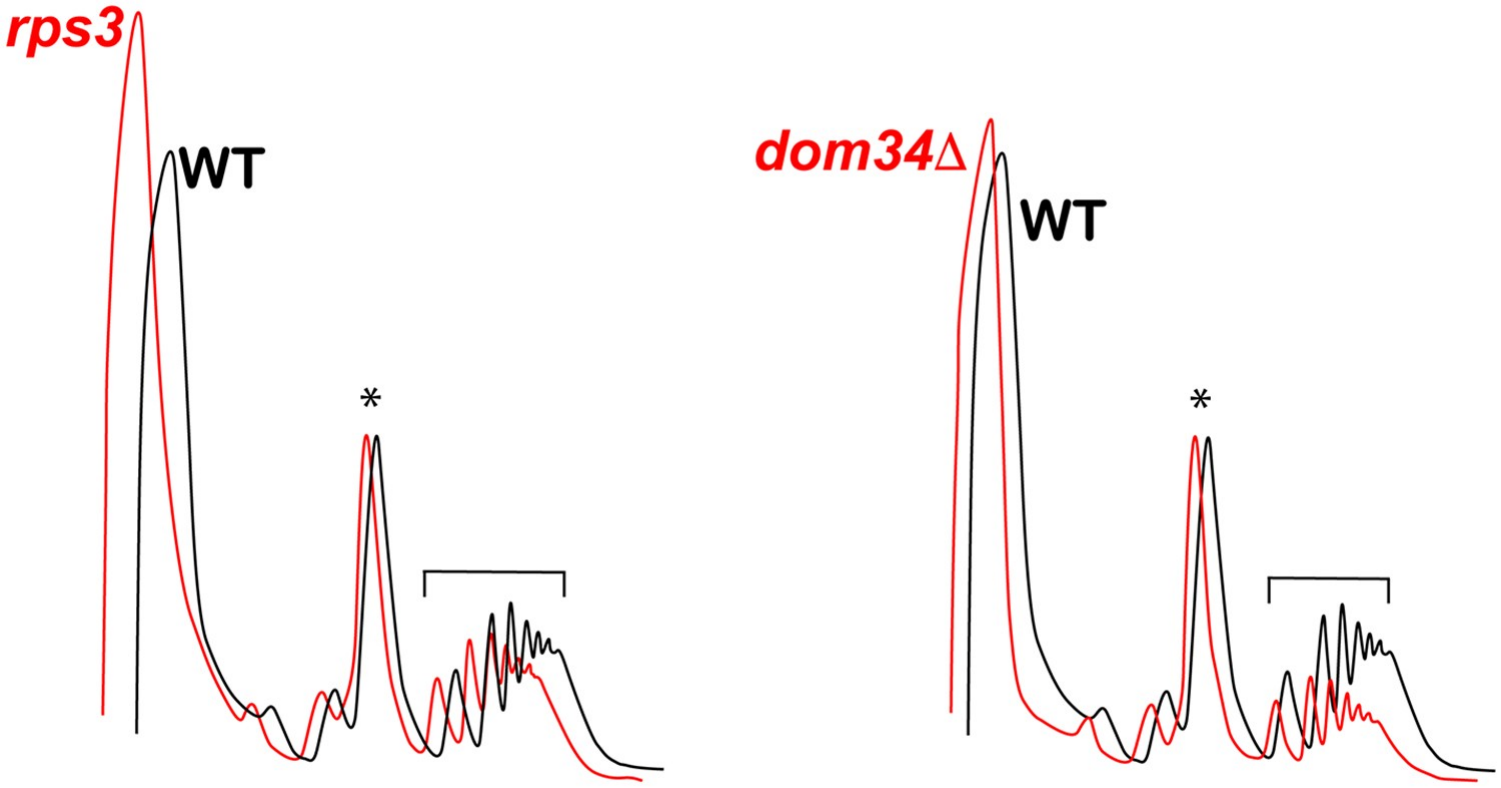
Mutations in Rps3 do not affect global ribosome density. Polysome profiles from wild type cells (black) and either *rps3* or *dom34Δ* mutant cells (red). The ratio of peak height for polysomes (bracket) to monosomes (asterisk) in *rps3* cells is similar to wild type (left) while this ratio is substantially lower in *dom34Δ* cells (right).

Recently we showed that ribosome collision appears to play an important role in initiating NGD during stalling [17]. In particular, decreasing ribosome concentration, and hence ribosome density per mRNA, by deleting certain ribosomal protein paralogues was found to reduce cleavage of NGD targets [40]. As a result, we wondered whether the mutations of the entry tunnel residues had similar effects on ribosome density. To address this potential explanation, we compared the polysome profile of the *rps3* cells to the wild-type ones. Our analysis revealed that the mutations in *RPS3* had little effect on ribosome density (Fig 6). The ratio of polysomes to monosomes in the mutant is largely similar to that observed in the wild-type background. In contrast, similar analysis of the *dom34Δ* cells- as has been seen before [27]- revealed elevated levels of 80S monosomes relative to polysomes (Fig 6). The finding that the *RPS3* mutations do not seem to affect ribosome density has two immediate ramifications: 1) the observed inhibition of NGD in the presence of these mutations does not result from changes to ribosome collisions; 2) consistent with our mapping analysis, the mutations are not likely affecting the function of Dom34.

### Stalling-induced ubiquitination of ribosomal proteins is unchanged in the presence of the R116A/R117A mutations

**Fig 7:**
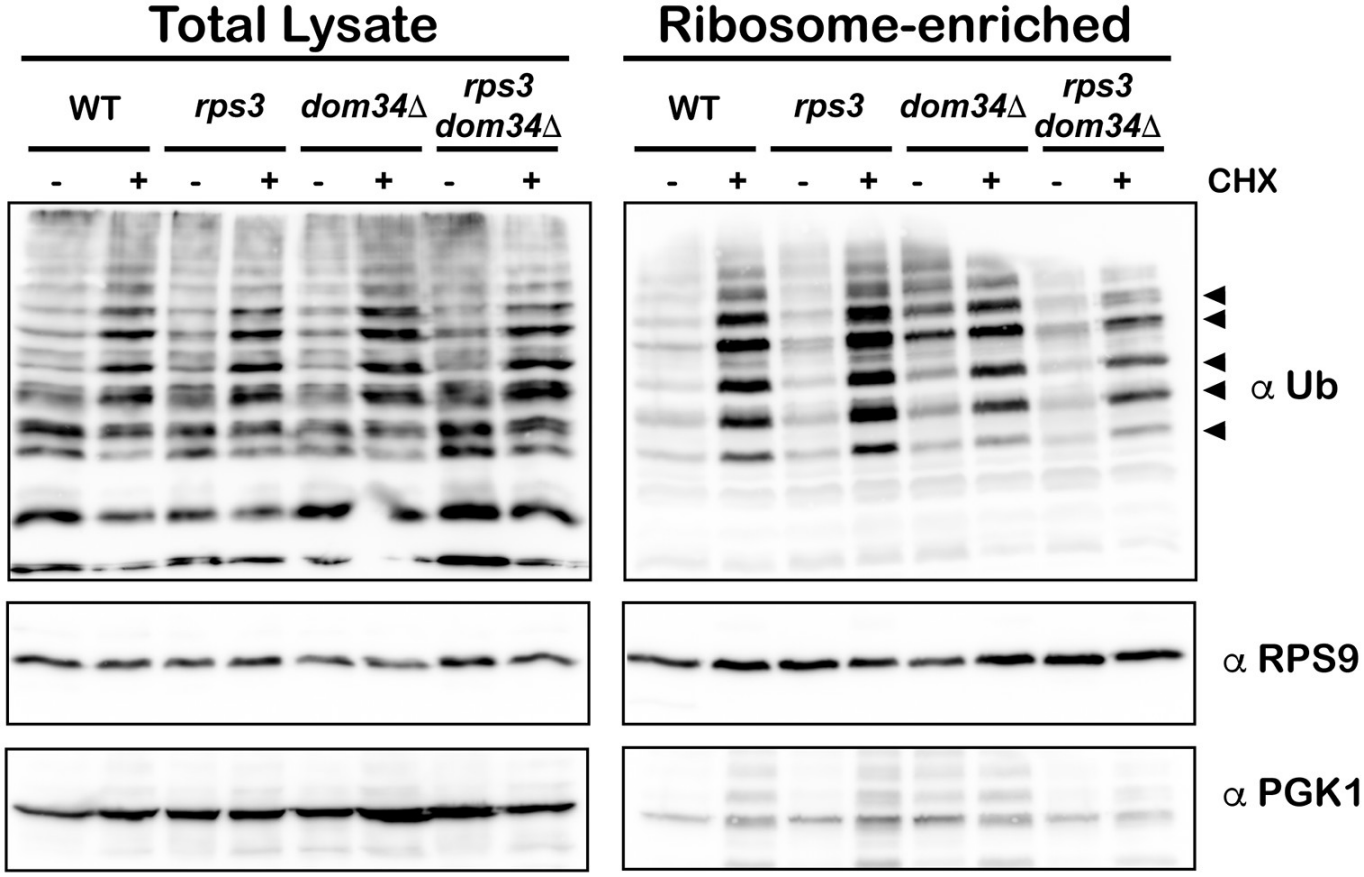
Ubiquitination of ribosomal proteins upon stalling is not affected by *rps3* mutations. Western blot of total cell lysate (left) or ribosome-enriched lysates (right) from the indicated strains. Cells were treated with cycloheximide at 2μg/mL to induce ribosome stalling. Ubiquitination patterns were essentially the same in cells with *rps3* R116A/R117A compared to those with wild type Rps3. Top panel shows blot for anti-ubiquitin; middle and bottom panels are blotted for Rps9 and Pgk1, respectively, as controls.

As discussed earlier, ubiquitination of ribosomal proteins by Hel2 (Znf598 in humans) has recently been recognized as an important feature of ribosome stalling. This modification promotes stalling on inhibitory codons as deletion of *HEL2* results in significant bypassing of stalls by the ribosome [36–38, 63, 64]. Relevant to our studies is the observation that Rps3 is one of the targets for Hel2-mediated ubiquitination on K212, but it is currently unclear if its modification is important for stalling [64]. Nevertheless, if the entry tunnel mutations somehow affect Hel2 function, this could in principle explain their effect on NGD. As a result, we set out to assess stalling-induced ribosomal protein ubiquitination in the presence of R116A/R117A mutations. We took advantage of our previous observation that the addition of cycloheximide to an intermediary concentration, whereby ribosome collisions presumably occur at a global level, results in robust ribosomal protein ubiquitination [62]. We added cycloheximide to a final concentration of 2 μg/mL to wild-type, *rps3* R116A/R117A, *dom34Δ* and double mutant cells; and isolated ribosomes. Ubiquitination patterns of ribosomal proteins resulting from cycloheximide addition, as assessed by western-blotting, was nearly identical among all strains (Fig 7). However, we noted that deletion of *DOM34* had a discernible effect on the ubiquitination levels suggesting that Dom34 might affect Hel2 function (Fig 7). The *rps3* mutations on their own, however, had no observable effect on the efficiency of ribosomal proteins ubiquitination. Hence, it is very unlikely that the effect of the entry-tunnel mutations on NGD are due to differences in ribosomal protein ubiquitination during stalling.

### *RPS3* mutations render cells sensitive to cycloheximide and RNA-damaging agents

**Fig 8:**
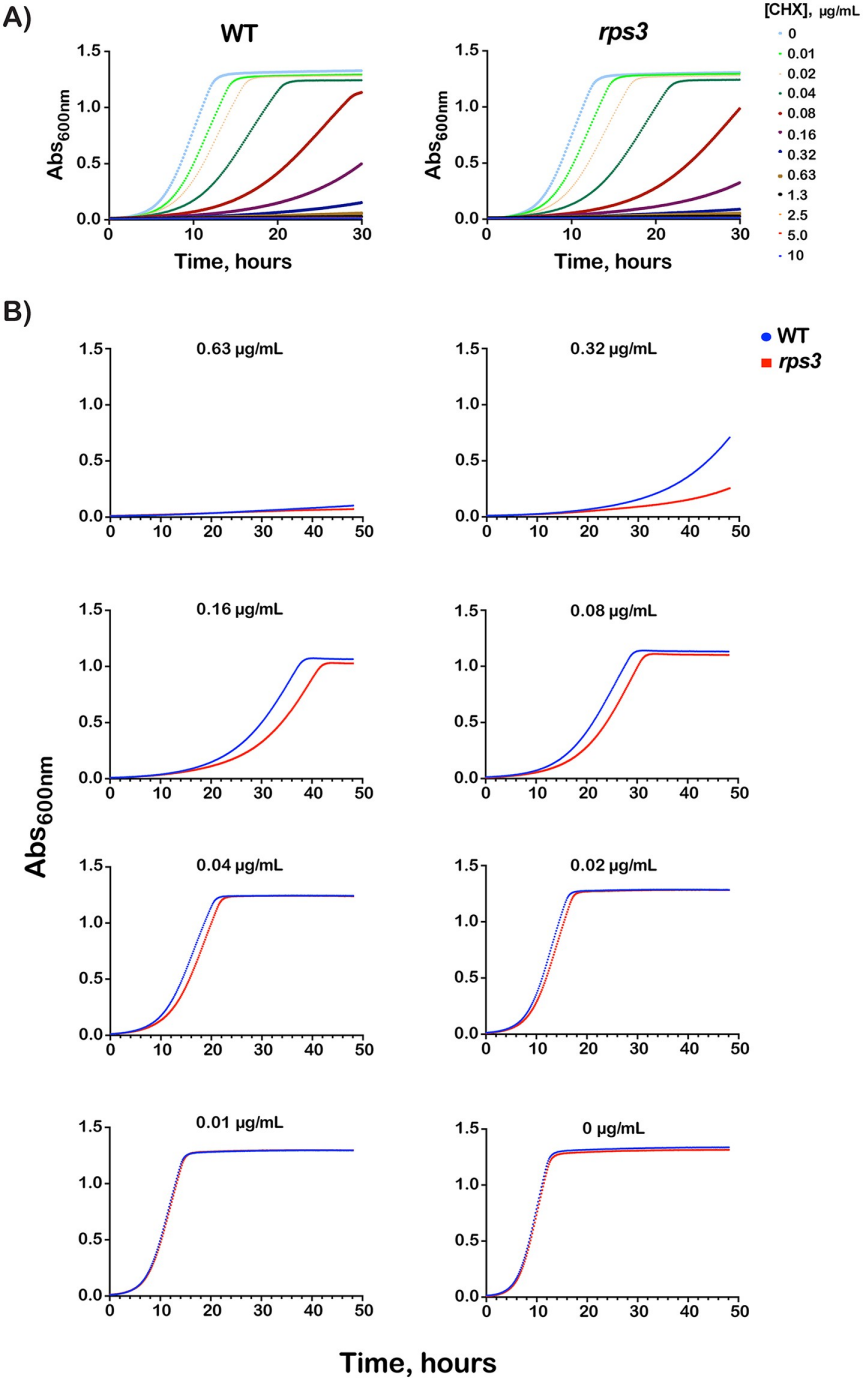
*RPS3* mutations result in increased sensitivity to cycloheximide. (**A**) Plot of OD_600_ over time of wild type or *rps3* mutant cells grown in the presence of cycloheximide at the indicated concentrations. (**B**) Individual plots from (**A**) showing data at the specified cycloheximide concentration. *rps3* mutations affect growth at intermediate concentrations of cycloheximide. Data was collected in technical duplicates from three biological replicates.

We reasoned that if the entry-tunnel residues of Rps3 are affecting NGD, then mutating them should result in increased sensitivity to cycloheximide especially at intermediate concentrations, at which ribosome collisions will occur and hence NGD is triggered. Growth of the *rps3* strain was compared to the wild-type one in the presence of varying concentrations of cycloheximide (Fig 8A and 8B). To distinguish between effects on the growth rate versus lag time, we determined the first derivative of the growth curve to measure the instantaneous growth rate. The maxima of the resulting curves report on the maximal growth rate, whereas the distance between the maxima reports on the lag. As expected, the mutations had no effect on the growth rate or lag period in the absence of the drug and at very low and high concentrations (S3 Fig). In contrast and in agreement with our model, the addition of cycloheximide at intermediate concentrations (0.02–0.32 μg/mL) significantly increased the lag period for the R116A/R117A mutant. This effect was most noticeable at the 0.16 μg/mL concentration, for which we observed a lag-time difference between the wild-type and the mutant cells of more than 4 hours (S3 Fig). Our data suggests that the entry-tunnel residues are important for dealing with intermittent collision events, and likely the ensuing process of ribosome rescue.

**Fig 9:**
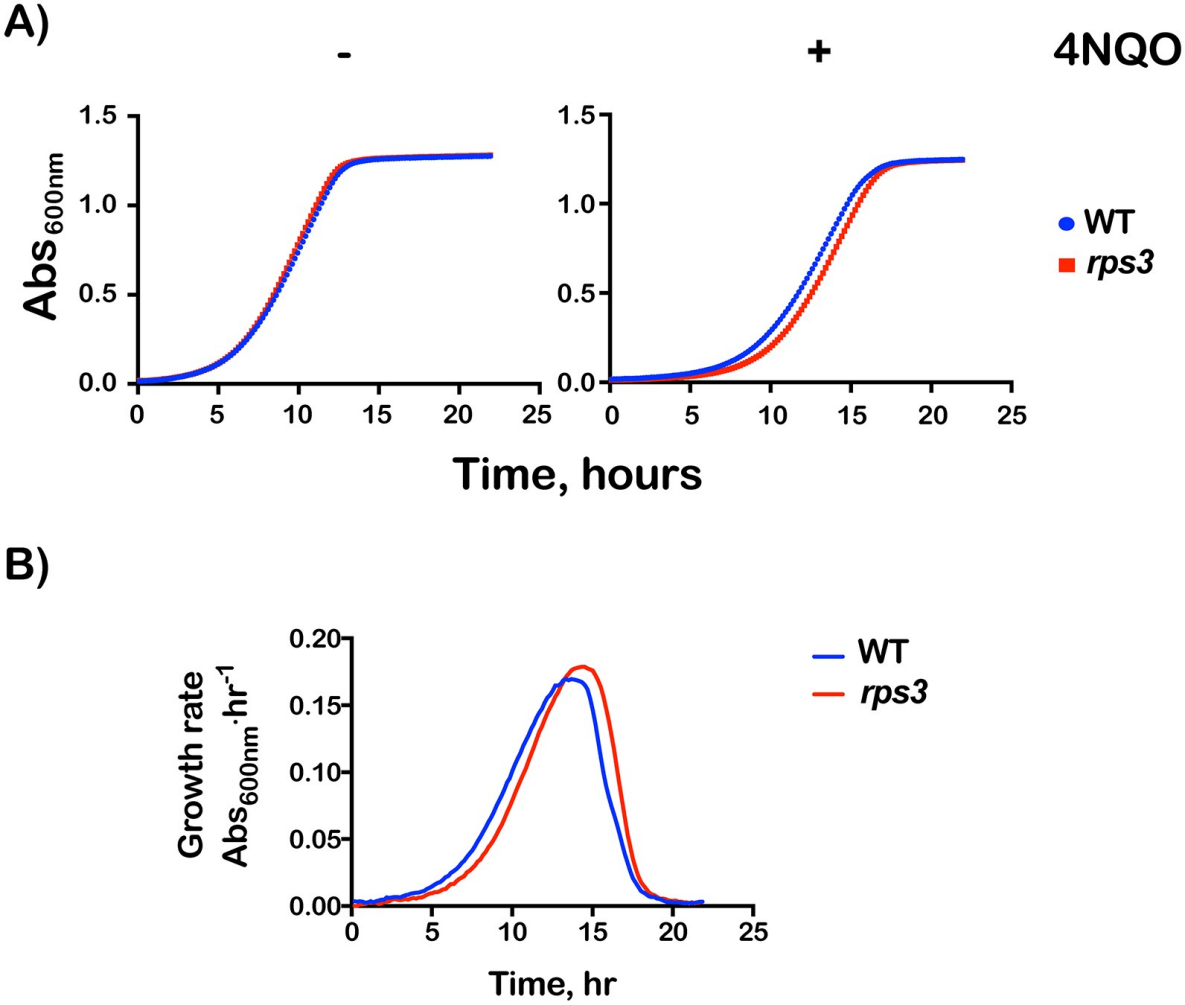
Mutations in Rps3 render cells sensitive to 4-NQO. (**A**) Plot of OD_600_ over time for wild type (blue) and rps3 cells (red) in the absence or presence of 4-nitroquinoline oxide. (**B**) Instantaneous growth rate plot for samples in (**A**) treated with 4-NQO indicates increased lag time of recovery from the drug for the *rps3* mutant. Data was collected in technical duplicates from three biological replicates.

Previous work from our lab revealed that RNA oxidation strongly stalls translation *in vitro* [17]. In particular, the introduction of a single 8-oxoguanosine adduct to the mRNA reduced the rate of peptide-bond formation by almost three orders of magnitude in a bacterial reconstituted system and prevented the formation of full-length protein products in wheat-germ and rabbit-reticulocyte extracts. We also provided evidence that showed oxidized mRNA is subject to NGD. Because our *rps3* mutations appear to affect NGD, they should also in principle result in increased sensitivity to agents that react with RNA to produce adducts such as 8-oxoguanosine. We used the chemical 4-Nitroquinoline 1-oxide (4NQO), a UV mimetic and known to produce reactive oxygen species, to introduce 8-oxoguanosine into RNA [65] in living yeast. Wild-type and *rps3-*mutant cells were grown to mid-logarithmic before being challenged with 5 μg/mL of 4NQO for 30 minutes. Cells were washed with fresh media, diluted and their growth monitored. In the absence of any drugs, the *rps3* mutant displayed a growth rate nearly identical to that of the wild-type (6.6 ±0.23 versus 6.3 ±0.07 hours). After incubation with 4NQO, the mutant displayed a notable lag in its growth of 1.4 hours (10.4 ±0.18 versus 9.0 ±0.79) (Fig 9A and 9B). We note that although the effects we saw are modest, they are reproducible and suggest that mutations of the entry-tunnel residues render cells sensitive to damaging agents. These effects are also reminiscent of the effects that we and others have documented for *dom34Δ* and *xrn1Δ* strains [17]. These findings together with the observation that mutations in *RPS3* result in increased sensitivity towards cycloheximide provide further support for a role for the factor in NGD.

## Discussion

NGD is a conserved eukaryotic process that responds to stalled ribosomes [14]. The process is characterized by an endonucleolytic cleavage of the aberrant mRNA upstream of the lead ribosome [15] and as yet the identity of the culprit endonuclease remains unknown. As a result, there is a critical gap in our understanding of some of the mechanistic details of the process. Nonetheless, multiple studies have provided important hints about the enzyme. For instance, mapping experiments suggested that the endonuclease is ribosome-associated [40, 41]. In particular, cleavage takes place in frame with the ribosome and is phased by ~30 nt, the mRNA-length protected by the ribosome. Furthermore, the reaction appears to likely take place between stacked ribosomes [40]. These studies hinted at a role for the ribosome itself in activating or recruiting the endonuclease. Here we provided further evidence for this notion. More specifically, we find the entry tunnel of the ribosomal protein Rps3 to be important for the cleavage reaction. Mutation of the key-entry-tunnel residues Arg116 and Arg117 were found to drastically affect the outcome of the cleavage event; we observe a significant reduction in the accumulation of 5’-fragments from a number of NGD reporters when these residues are mutated to Ala. Consistent with these findings, although subtle, the half-life of the SL reporter increases in the presence of the mutations suggesting that these mutations may stabilize NGD reporters. Mapping of the cleavage products also revealed spreading of the cleavage reaction in the presence of the mutations. We note that Rps3 is known to interact with two key NGD factors: Dom34 and ribosome-associated Asc1 [60, 66]. Although deletion or mutation of these factors affects the cleavage pattern in the *rps3* background, as evidenced by northern analysis, the effect of the mutations on NGD do not appear to phenocopy those observed in the *dom34Δ* and *asc1* strains, which is apparent in the high-throughput mapping data. Furthermore, the mutations do not alter Asc1 occupancy on the ribosome. Collectively our data suggests that the entry-tunnel region of Rps3, and hence the ribosome, has a function in NGD upon stalling. In agreement with this proposal, mutations of this region render cells sensitive to intermediate concentrations of cycloheximide and the nucleic-acid damaging agent 4NQO; both stall the ribosome and likely trigger NGD.

Apart from the decoding center nucleotides, the Arg116 and Arg117 residues of the entry tunnel of the ribosome come closest to the mRNA. Indeed, some of the first studies on this region showed it to be important for unwinding the mRNA and make up part of the helicase domain of the ribosome [42]. While our data do not show the residues to be required for cleavage to take place–we still observe accumulation of NGD fragments in the presence of the mutations–they clearly affect the pattern of the cleavage reaction. It is feasible that the electrostatic interaction between the side chains and the phosphodiester backbone of the mRNA is important for locking the mRNA in place for the endonuclease to carry out its cleavage reaction. When these residues are mutated to Ala residues, the mRNA is more dynamic and its accessibility to the enzyme’s active site is severely affected. Alternatively, these residues might be important for recruiting or activating the endonuclease and as a result, changing their identities inhibits the cleavage reaction, although it is not clear how residues buried deep in the ribosome could be used efficiently to recruit exogenous protein factors. Instead, we favor a model whereby the endonuclease is intimately associated with the ribosome and it is activated upon stalling. In agreement with this, previous work has indicated that during non-stop decay, when the ribosome runs to the end of an mRNA, the endonucleolytic cleavage takes place near the exit tunnel of the ribosome [16, 41, 67] as evidenced partly by the accumulation of 15–18 nt fragments. Similarly, during a novel form of mRNA degradation termed ribothrypsis, it was suggested that an endonucleolytic cleavage event takes place near the exit tunnel [68]. Interestingly, recent structural data from human cells has revealed the position of multiple ribosomal proteins and associated factors at collided di-ribosomes–events that trigger NGD [69]. It appears that this higher order structure brings an entry- and exit-tunnel face of adjacent ribosomes in close proximity, which could potentially allow for interactions between otherwise distally positioned components. These include RACK1/Asc1 on the stalled ribosome with uS3, eS10, and uS10 on the collided ribosome, as well as eS26 and eS28 facing uS4 and rRNA helix 16 on the stalled and collided ribosomes, respectively. It will be exciting to see how modifications to these factors may affect endonuclease activity.

In an endonuclease-independent consequence, the residues and their interaction with the mRNA could play a role in recruiting Dom34 and Hbs1 to the ribosome. Biochemical and structural studies have suggested that Hbs1 is recruited to a ribosome with little to no mRNA downstream of the A site [20, 21, 66, 70]. The N-terminal of Hbs1 binds in the RNA entry tunnel, interacting with Rps3 [66]. It was hypothesized that Hbs1 cannot bind in the presence of mRNA in the entry tunnel [19, 20, 60, 66]. Additional recent structural studies also revealed a potential role for Dom34 in sensing the mRNA channel, whereby it uses a unique β-loop to protrude into the mRNA channel to sense its absence [60]. Together these two mechanisms ensure that ribosome dissociation only occurs when the ribosome reaches the end of the mRNA, such as during NSD or on the behind ribosomes following cleavage during NGD. It is possible that the mutations in the entry tunnel of Rps3 make the mRNA more dynamic, preventing a clash with Dom34 and Hbs1. In turn, this allows the factors to bind and dissociate the ribosomes before cleavage could take place. In agreement with this model, deletion of *DOM34* in the presence of the *rps3* mutations restores cleavage efficiency, and with increased heterogeneity, as expected, due to widespread ribosome queueing. This model, however, does not explain why the cleavage patterns in the double mutant do not look similar to those observed in the *dom34Δ* mutant. Therefore, the effects of the *rps3* mutations appear to be more complex and they are likely to alter different aspects of NGD including the cleavage and the dissociation reactions. In contrast, the mutations do not appear to affect the RQC pathway, as we observe comparable ribosomal protein ubiquitination patterning and efficiency upon inducing ribosome collisions regardless of the status of Rps3.

Perhaps not surprising given its proximity to the mRNA, Rps3 plays a number of roles on the ribosome during translation. It has been shown to be important for providing the helicase activity to the ribosome; in bacteria Rps3/uS3, together with Rps4/uS4 and Rps5/uS5, encircle the incoming mRNA within the entry tunnel. When Arg131 and Arg132 in bacteria (corresponding to Arg116 and Arg117 in yeast) were mutated to Alanine, the efficiency of unwinding an RNA duplex by the ribosome was reduced [42]. Residues of Rps4 were also shown to contribute to helicase activity, but the process overall is coupled to and dependent on movements during translocation [71]. Rps3 is known to interact with other ribosomal proteins, including ribosome-bound Asc1/RACK1 [60, 66]. In addition to its aforementioned role in NGD, Asc1 is known to be involved in preventing readthrough of inhibitory codons and reading-frame maintenance [72]. In eukaryotes, the C-terminal tail of Rps3 lies further inside the mRNA channel, proximal to Asc1 [43]. It is tempting to speculate that conformational changes that involve Rps3 could be communicated to Asc1, which then may initiate additional steps in NGD. However, the convergence of phenotypes among Rps3, Asc1 and Dom34 highlight the potential for redundancy or simply subtle differences of function between these and related factors. This is also evident during non-functional 18S rRNA decay (NRD), where both Asc1 and Rps3 have recently been identified as players in the pathway [73]. The post-translationally modified C-terminal tail of Rps3 is required for 18S NRD and, as Asc1 can collaborate with either Dom34 or Hbs1, it was suggested that multiple overlapping pathways function to deal with damaged rRNA. At another step in the translation cycle, Rps3 also contributes to stabilizing the incoming mRNA during initiation. Again, yeast residues Arg116 and Arg117 were shown to promote binding of the mRNA to eIF3 dependent pre-initiation complexes (PICs) and in particular, when the exit channel is empty, they were absolutely required [46]. This demonstrates the diverse functionality of Rps3 that is likely due in part to its position at the entry tunnel where it interacts with and can survey incoming transcripts.

Collectively our findings provide further evidence for the central role of the ribosome in mRNA-surveillance pathways beyond just recognizing the aberrant mRNA and initiating the downstream events. The observation that mutations deep into the ribosome lead to dramatic changes to NGD bolsters arguments by us and others that the endonuclease is likely to be an integral part of the machine. This in turn could explain why it has been difficult to identify the endonuclease. It would be interesting to examine how quality control mechanisms evolved to integrate into fundamental biological machines. Further delineation of the details of this mechanism will also contribute to the understanding of how cells identify and degrade defective biological molecules. Finally, similar to NMD, NGD is likely to have been coopted to regulate gene expression. Indeed, recent reports have shown conditional deletion of Pelota (the human orthologue of Dom34) results in abnormal cellular differentiation [74]. The identification of the endonuclease is more than likely to provide further and important appreciation of the pervasiveness of this mode of gene regulation through NGD.

## Methods

### Yeast strains and plasmids

Cells were grown at 30°C in YPD or in defined media when expressing reporter plasmids. Yeast strains were made using standard PCR-based disruption techniques in the background BY4741 (*MATa* (*his3Δ1 leu2Δ0 met15Δ0 ura3Δ0*). SKI7 knockout strains were generated with a LEU2 cassette, amplified using oligos complementary to the insertion site.

RPS3 mutant strains were constructed by first cloning a fragment encoding RPS3-HIS3-rpS3 3’UTR, generated by fusion PCR, into the BamHI/XhoI sites in pPROEX-HTb. Point mutations in RPS3 were introduced by site directed mutagenesis and a cassette encoding the entire region was PCR amplified and used to transform the target yeast strains. RPS2 (E120A) strains were made using the same method and ASC1 (R38D, K40E) strains were made similarly, except using BamHI/XbaI sites in pET28a. HIS3 and LEU2 coding regions were amplified from plasmids pFAGa-6xGLY-FLAG-HIS3 and pAG415 [75] respectively.

Plasmids encoding the PGK1 gene or PGK1-SL under control of the GAL1 promoter were obtained from R. Parker [15]. PGK1-(CGA)_12_, PGK1-(AAA)_12_ and PGK1-(UUU)_12_ were made by annealing complementary oligos and ligating them to XbaI digested PGK1 plasmid [40].

### Northern blotting

Culture was grown overnight in a defined media (-Ura) with glucose. Cells were washed twice in media containing 2% Raffinose and 2% galactose, diluted to OD 0.1 in the same media and grown to an OD of 0.5–0.8 to permit expression of the gal-driven reporters. RNA was isolated using hot phenol extraction followed by two sets of chloroform extraction and ethanol precipitation. 2μg of total RNA was resolved on 1.2% formaldehyde agarose gel, followed by transfer to positively-charged nylon membrane (GE Lifesciences) using a vacuum blotter (Biorad). Next, nucleic acids were UV cross-linked to the membrane and baked at 80°C for 15 minutes. Membranes were then pre-hybridized in Rapid-Hyb buffer (GE Lifesciences) for 30 minutes in a hybridization oven. Radiolabeled DNA probe, which was labeled using polynucleotide kinase and [γ-^32^P]ATP, was added to the buffer and incubated overnight. Membranes were washed with nonstringent buffer (2 × SSC, 0.1% SDS) three times, in some cases followed by three washes in stringent buffer (0.2 × SSC, 0.1% SDS), all at hybridization temperature. Membranes were exposed to a phosphorimager screen and analyzed using a Biorad Personal Molecular Imager.

All Northern analyses were performed using at least three biological replicates. Representative images are shown.

### RNA half-life measurements

Cells expressing PGK1-SL were grown overnight in defined media (-Ura) plus glucose. Cultures were then washed in -Ura media, resuspended at OD 0.1 in 50 mL -Ura plus galactose, and grown for 18–20 hours to allow expression of the reporter plasmid. Cells were collected at OD 0.5–0.6, washed once and resuspended in 11 mL pre-warmed -Ura media. A 1 mL aliquot was saved for the t_0_ timepoint and 1 mL 40% glucose added to the remainder. Cells were incubated at 30°C while shaking and aliquots taken at the indicated timepoints. For each sample, cells were pelleted, media was removed, and tubes were frozen on dry ice. RNA was isolated using a hot phenol method followed by two rounds of chloroform extraction and ethanol precipitation. 2 μg of total RNA for each sample was analyzed by Northern blot.

### Polysomes analysis

Yeast cultures were grown to mid-log phase before addition of cycloheximide to a final concentration of 100 μg/mL. The culture was chilled by adding an equal volume of ice and centrifuged at 4°C. Cells were then resuspended in polysome lysis buffer (20 mM Tris pH 7.5, 140 mM KCl, 5 mM MgCl_2_, 0.5 mM DTT, 1% Triton-100, 100 μg/mL cycloheximide, 200 μg/mL heparin), washed once and lysed with glass beads using a FastPrep (MP Biomedical). Supernatant from cleared lysate corresponding to 1 mg of total RNA was layered over a 10–50% sucrose gradient and centrifuged at 37,000 rpm for 160 min in an SW41Ti (Beckman) swinging bucket rotor. Gradients were fractionated using a Brandel tube-piercing system combined with continuous absorbance reading at A_254_ nm. Proteins were precipitated by the addition of TCA to 10% after a twofold dilution with water, and resuspended in HU buffer (8 M Urea, 5% SDS, 200 mM Tris pH 6.8, 100 mM DTT).

### Western blotting

Proteins were resolved on 15% SDS PAGE gels and transferred to PVDF membranes using a semi-dry transfer apparatus (BioRad). The membranes were blocked with milk in PBST for ~ 30 minutes at room temperature followed by incubation with primary antibody overnight at 4°C. After washing with PBST, the membrane was incubated with the appropriate HRP-conjugated secondary antibody for ~ 1hr at room temperature before washing 3–4 × with PBST. Detection was carried out on a GE ImageQuant LAS 4000 using the Pierce SuperSignal West Pico Chemiluminescent Substrate. The following antibodies were used: mouse anti-PGK1[22C5D8] (ab113687) and rabbit anti-rpS9 (ab117861) from Abcam; rabbit anti-ASC1 was a gift from Wendy Gilbert (Yale University) [61]; mouse anti-rpL4 was a gift from Heather True (Washington University in St. Louis); goat anti-mouse IgG HRP (31430) and goat anti-rabbit IgG HRP (31460) from Thermo Scientific.

### High throughput sequencing

Total RNA from the indicated strains was ligated to a short adenylated DNA oligonucleotide, 5'rAppCTGTAGGCACCATCAAT/3ddC/ 3', at its 3’ end using truncated T4 RNA ligase 2 (NEB). For each sample, total RNA from at least two biological replicates was included. Reverse transcription using a primer complementary to the adaptor was performed, and then cDNA was amplified with a 5’-primer that annealed at position 585 of PGK1. Primers were designed for the Illumina HiSeq platform and samples were column purified to remove primers before sequencing.

Single-read HiSeq 2500 sequencing was performed by the Genome TechnologyAccess Center (GTAC) at Washington University. Raw data was analyzed for quality using the Fastx toolkit (http://hannonlab.cshl.edu/fastx_toolkit/index.html), trimmed using cutadapt [76] and aligned to our reference reporter sequence using NovoAlign (http://www.novocraft.com/). Sequencing results are available at GEO (accession #GSE117652).

### Growth curves and sensitivity assays

Sensitivity assays were conducted essentially as described [74]. Yeast cells were grown to mid-log-phase (OD_600_ of 0.5–0.7), collected, washed and resuspended in YPD to a final density of OD 0.8. 5 μl of the cell suspension was added to 195 μl of YPD with CHX at various concentrations, from 0–10 μg/mL. All samples were prepared in biological triplicates as well as technical duplicates in 96-well polystyrene microplates. The plate was incubated at 30°C with shaking on a microplate scanning spectrophotometer (Biotek). Cell density was monitored every 10 min over 24–48 h at 600nm. To assay sensitivity to 4NQO, after growing cells to mid-log (OD 0.5–0.7) cultures were treated with and without 5 μg/mL 4QNO for 30 minutes. Cells were collected, washed and adjusted to OD 0.8. Samples were plated and growth monitored as above.

## Supporting information

S1 Fig Deletion of *SKI7* or mutations in *RPS2* do not modify the effect of *RPS3* mutation on cleavage. (**A**) Northern analysis of 5’ fragments generated in the indicated strains, each with and without *ski7Δ* and mutations in *RPS3*. Deletion of *SKI7* does not affect the cleavage reaction when paired with *rps3* mutations. (**B**) Northern analysis of 5’ fragments accumulation from cells with and without mutations in *RPS3* and *RPS2*. In both A and B, bottom panels are the corresponding ethidium-bromide stained agarose gels.
(TIF)

S2 Fig Large-scale sequencing of (CGA)_12_ and (AAA)_12_ reporters. Plots of sequencing reads from the indicated strains expressing either a (CGA)_12_ reporter (**A**) or a (AAA)12 reporter (**B**). Each point represents a single read, mapped relative to the distance upstream of the stall site. Top panels are from wild type cells, middle panels from *rps3* R116A/R117A and bottom panels are from *dom34Δ* cells. In all cases, strains are in a *ski2Δ* background and data from wild type panels is adapted from Simms et al [62].
(TIF)

S3 Fig Instantaneous growth rates in the presence of cycloheximide. First derivative of growth curves from Fig 9B, with wild type cells shown in blue and *rps3* R116A/R117A cells shown in red. Cells were grown at the indicated cycloheximide concentrations–intermediate concentrations result in increased lag time as indicated by shift between the maximas. Data was collected in technical duplicate from three biological replicates.
(TIF)

S1 Table List of yeast strains. (PDF)

S2 Table List of DNA oligos. (PDF)

S1 Dataset Data for RNA turnover experiments. (XLSX)
