# Supplementary figures and images for: Interactions between the mRNA and Rps3/uS3 at the entry tunnel of the ribosomal small subunit are important for no-go decay
Source: PLoS Genet. 2018 Nov 26;14(11):e1007818. doi: 10.1371/journal.pgen.1007818 (PMC6283612; doi:10.1371/journal.pgen.1007818)

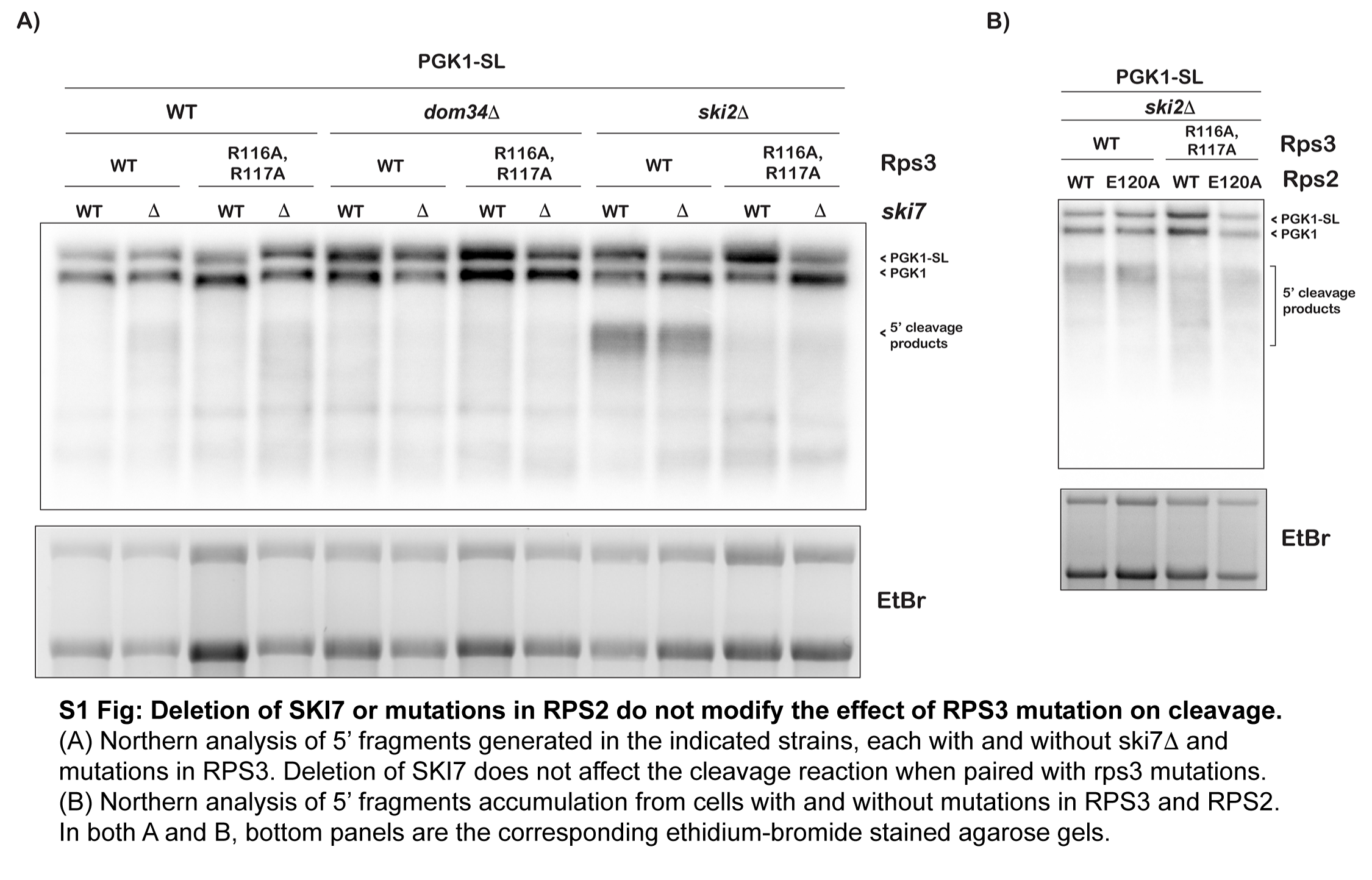

Supplement: S1 Fig — (A) Northern analysis of 5’ fragments generated in the indicated strains, each with and without ski7Δ and mutations in RPS3. Deletion of SKI7 does not affect the cleavage reaction when paired with rps3 mutations. (B) Northern analysis of 5’ fragments accumulation from cells with and without mutations in RPS3 and RPS2. In both A and B, bottom panels are the corresponding ethidium-bromide stained agarose gels. (TIF) [file pgen.1007818.s001.tif]

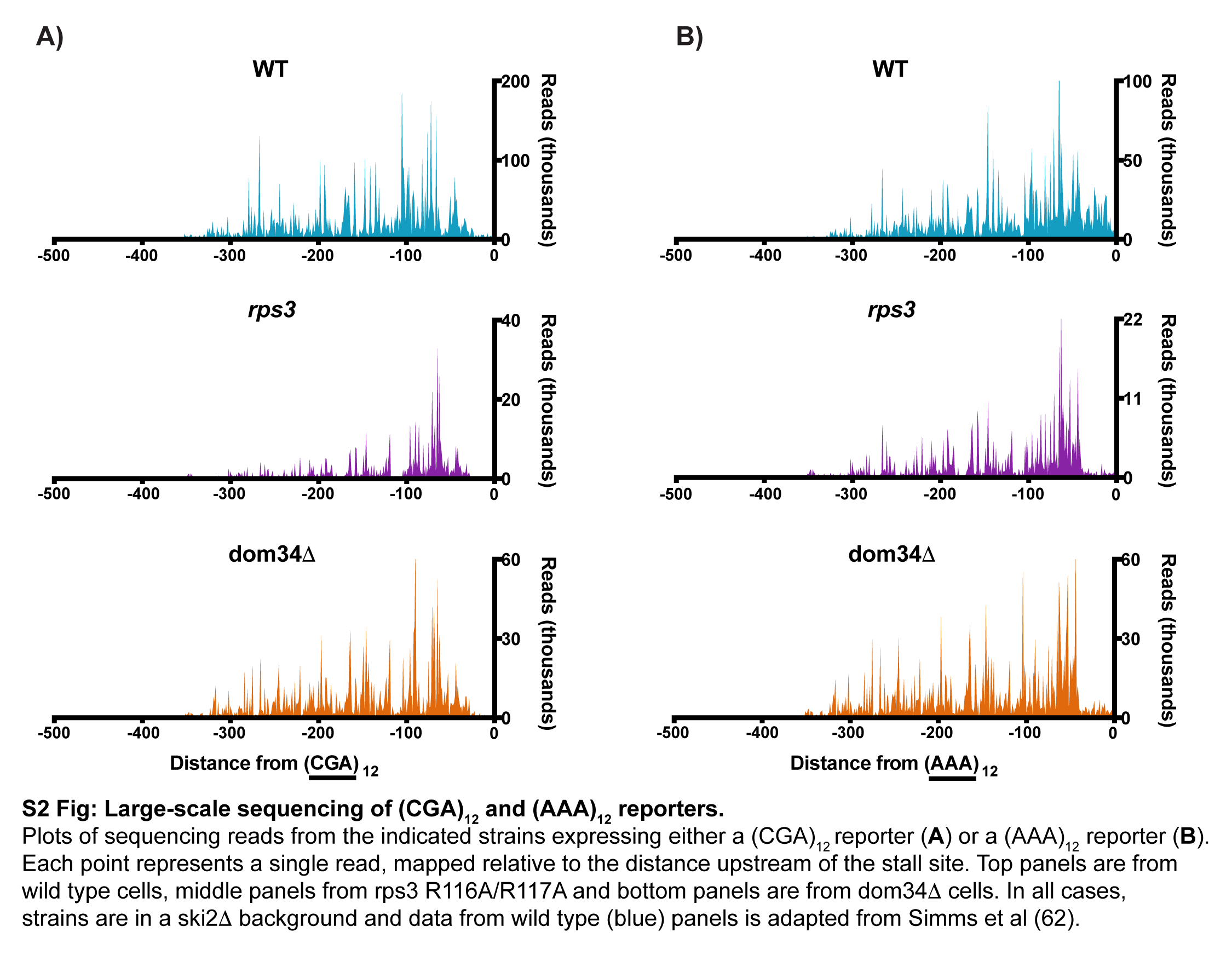

Supplement: S2 Fig — Plots of sequencing reads from the indicated strains expressing either a (CGA)12 reporter (A) or a (AAA)12 reporter (B). Each point represents a single read, mapped relative to the distance upstream of the stall site. Top panels are from wild type cells, middle panels from rps3 R116A/R117A and bottom panels are from dom34Δ cells. In all cases, strains are in a ski2Δ background and data from wild type panels is adapted from Simms et al [62]. (TIF) [file pgen.1007818.s002.tif]

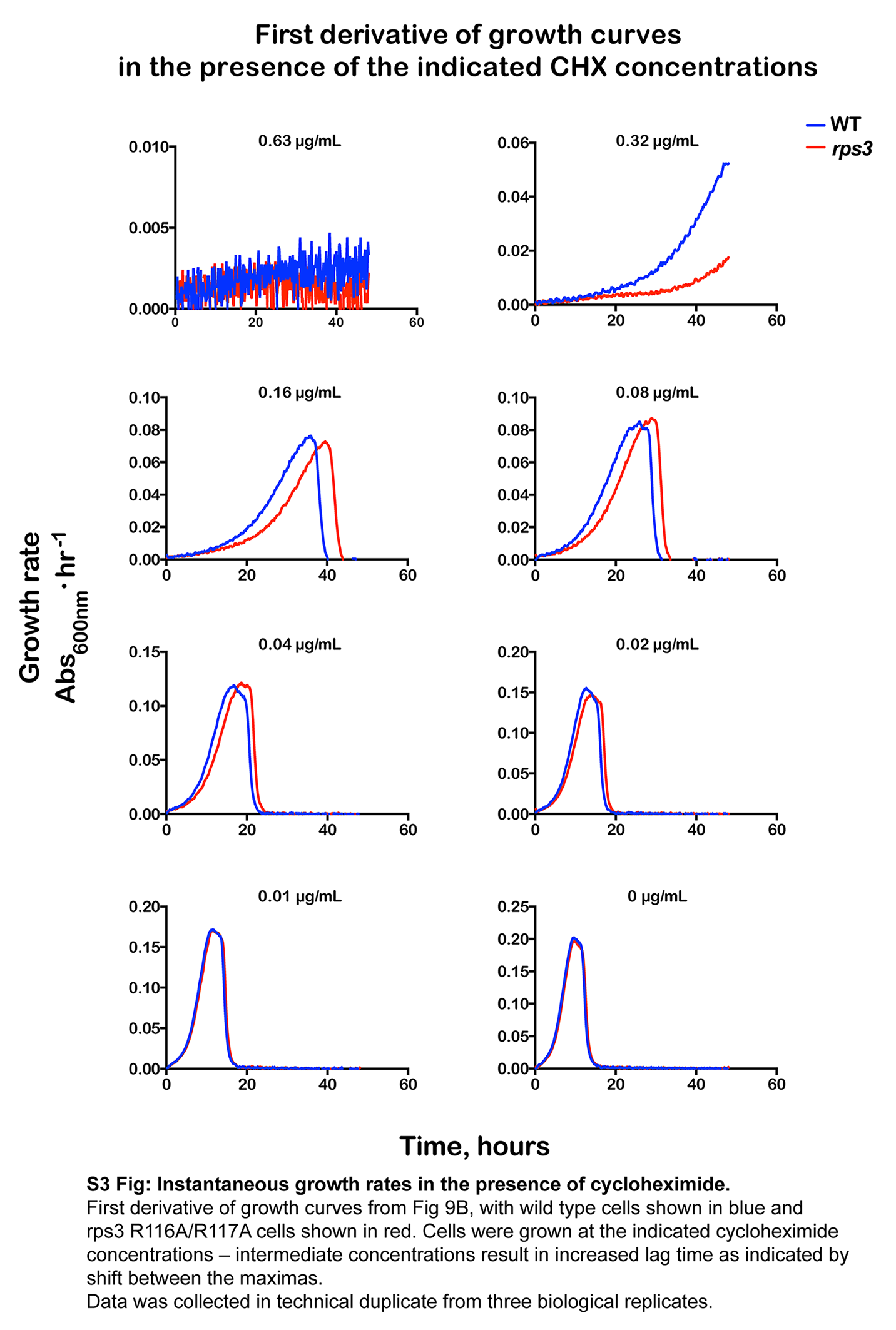

Supplement: S3 Fig — First derivative of growth curves from Fig 9B, with wild type cells shown in blue and rps3 R116A/R117A cells shown in red. Cells were grown at the indicated cycloheximide concentrations–intermediate concentrations result in increased lag time as indicated by shift between the maximas. Data was collected in technical duplicate from three biological replicates. (TIF) [file pgen.1007818.s003.tif]
