# Supplementary material for: Interactions between the mRNA and Rps3/uS3 at the entry tunnel of the ribosomal small subunit are important for no-go decay
Source: PLoS Genet. 2018 Nov 26;14(11):e1007818. doi: 10.1371/journal.pgen.1007818 (PMC6283612; doi:10.1371/journal.pgen.1007818)
Supplement: S1 Table — (PDF) [file pgen.1007818.s004.pdf]

S1 Table: List of yeast strains used in this work

Strain	Genotype	Source
BY4741 (matA)	*MATa (his3Δ1 leu2Δ0 met15Δ0 ura3Δ0)*	Dharmacon
DOM34Δ	BY4741; dom34::KanMX	Dharmacon
XRN1Δ	BY4741; xrn1::KanMX	Dharmacon
SKI2Δ	BY4741; ski2::KanMX	Dharmacon
matA; RPS3	BY4741; rps3-HIS3	This work
DOM34Δ; RPS3	BY4741; dom34::KanMX; rps3-HIS3	This work
XRN1Δ; RPS3	BY4741; xrn1::KanMX; rps3-HIS3	This work
SKI2Δ; RPS3	BY4741; ski2::KanMX; rps3-HIS3	This work
matA; RPS3(R116A,R117A)	BY4741; rps3 (R116A, R117A)-HIS3	This work
DOM34Δ; RPS3(R116A,R117A)	BY4741; dom34::KanMX; rps3 (R116A, R117A)-HIS3	This work
XRN1Δ; RPS3(R116A,R117A)	BY4741; xrn1::KanMX; rps3 (R116A, R117A)-HIS3	This work
SKI2Δ; RPS3(R116A,R117A)	BY4741; ski2::KanMX; rps3 (R116A, R117A)-HIS3	This work
SKI2Δ; RPS3(D154A)	BY4741; ski2::KanMX; rps3 (D154A)-HIS3	This work
SKI2Δ; RPS3(K200N)	BY4741; ski2::KanMX; rps3 (K200N)-HIS3	This work
SKI2Δ; RPS3(D154A,K200N)	BY4741; ski2::KanMX; rps3 (D154A, K200N)-HIS3	This work
DOM34Δ; SKI2Δ; RPS3	BY4741; dom34::KanMX; ski2::KanMX; rps3-HIS3	This work
DOM34Δ; SKI2Δ; RPS3(R116A,R117A)	BY4741; dom34::KanMX; ski2::KanMX; rps3 (R116A, R117A)-HIS3	This work
matA; RPS3; ASC1	BY4741; rps3-HIS3; asc1-LEU2	This work
matA; RPS3(R116A,R117A); ASC1	BY4741; rps3 (R116A, R117A)-HIS3; asc1-LEU2	This work
matA; RPS3; ASC1(R38D,K40E)	BY4741; rps3-HIS3; asc1 (R38D, K40E)-LEU2	This work
matA; RPS3(R116A,R117A); ASC1(R38D,K40E)	BY4741; rps3 (R116A, R117A)-HIS3; asc1 (R38D, K40E)-LEU2	This work
SKI2Δ; RPS3; ASC1	BY4741; ski2::KanMX; rps3-HIS3; asc1-LEU2	This work
SKI2Δ; RPS3(R116A,R117A); ASC1	BY4741; ski2::KanMX; rps3 (R116A, R117A)-HIS3; asc1-LEU2	This work
SKI2Δ; RPS3; ASC1(R38D,K40E)	BY4741; ski2::KanMX; rps3-HIS3; asc1 (R38D, K40E)-LEU2	This work
SKI2Δ; RPS3(R116A,R117A); ASC1(R38D,K40E)	BY4741; ski2::KanMX; rps3 (R116A, R117A)-HIS3; asc1 (R38D, K40E)-LEU2	This work
SKI2Δ; RPS3; RPS2	BY4741; ski2::KanMX; rps3-HIS3; rps2-LEU2	This work

SKI2Δ; RPS3(R116A,R117A); RPS2	BY4741; ski2::KanMX; rps3 (R116A, R117A)-HIS3; rps2-LEU2	This work
SKI2Δ; RPS3; RPS2(E120A)	BY4741; ski2::KanMX; rps3-HIS3; rps2 (E120A)-LEU2	This work
SKI2Δ; RPS3(R116A,R117A); RPS2(E120A)	BY4741; ski2::KanMX; rps3(R116A, R117A)-HIS3; rps2 (E120A)-LEU2	This work
matA; RPS3; SKI7Δ	BY4741; rps3-HIS3; ski7::LEU2	This work
matA; RPS3(R116A,R117A); SKI7Δ	BY4741; rps3 (R116A, R117A)-HIS3; ski7::LEU2	This work
SKI2Δ; RPS3; SKI7Δ	BY4741; ski2::KanMX; rps3-HIS3; ski7::LEU2	This work
SKI2Δ; RPS3(R116A,R117A); SKI7Δ	BY4741; ski2::KanMX; rps3 (R116, R117A)-HIS3; ski7::LEU2	This work
DOM34Δ; RPS3; SKI7Δ	BY4741; dom34::KanMX; rps3-HIS3; ski7::LEU2	This work
DOM34Δ; RPS3(R116A,R117A); SKI7Δ	BY4741; dom34::KanMX; rps3 (R116A, R117A)-HIS3; ski7::LEU2	This work
