# Supplementary material for: Interactions between the mRNA and Rps3/uS3 at the entry tunnel of the ribosomal small subunit are important for no-go decay
Source: PLoS Genet. 2018 Nov 26;14(11):e1007818. doi: 10.1371/journal.pgen.1007818 (PMC6283612; doi:10.1371/journal.pgen.1007818)
Supplement: S2 Table — (PDF) [file pgen.1007818.s005.pdf]

S2 Table: DNA oligos used in this study.

Oligo	Purpose	Sequence
PGK-HS-RT3	HiSeq - amplify 5'fragments from RT reaction	AAT GAT ACG GCG ACC ACC GAG ATC TAC ACT CTT TCC CTA CAC GAC GCT CTT CCG ATC TAT TGA TGG TGC CTA CAG
HS-F2	HiSeq	CAA GCA GAA GAC GGC ATA CGA GAT ACT GAT GTG ACT GGA GTT CAG ACG TGT GCT CTT CCG ATC TGG TAA GGC TTT GGA GAA CCC A
HS-F3	HiSeq	CAA GCA GAA GAC GGC ATA CGA GAT ATG CTG GTG ACT GGA GTT CAG ACG TGT GCT CTT CCG ATC TGG TAA GGC TTT GGA GAA CCC A
HS-F4	HiSeq	CAA GCA GAA GAC GGC ATA CGA GAT ACG TCG GTG ACT GGA GTT CAG ACG TGT GCT CTT CCG ATC TGG TAA GGC TTT GGA GAA CCC A
HS-F5	HiSeq	CAA GCA GAA GAC GGC ATA CGA GAT AGC TGC GTG ACT GGA GTT CAG ACG TGT GCT CTT CCG ATC TGG TAA GGC TTT GGA GAA CCC A
HS-F6	HiSeq	CAA GCA GAA GAC GGC ATA CGA GAT ATC GTA GTG ACT GGA GTT CAG ACG TGT GCT CTT CCG ATC TGG TAA GGC TTT GGA GAA CCC A
HS-F7	HiSeq	CAA GCA GAA GAC GGC ATA CGA GAT TGG TCA GTG ACT GGA GTT CAG ACG TGT GCT CTT CCG ATC TGG TAA GGC TTT GGA GAA CCC A
HS-F8	HiSeq	CAA GCA GAA GAC GGC ATA CGA GAT CAC TGT GTG ACT GGA GTT CAG ACG TGT GCT CTT CCG ATC TGG TAA GGC TTT GGA GAA CCC A
HS-F9	HiSeq	CAA GCA GAA GAC GGC ATA CGA GAT ATT GGC GTG ACT GGA GTT CAG ACG TGT GCT CTT CCG ATC TGG TAA GGC TTT GGA GAA CCC A
HS-F10	HiSeq	CAA GCA GAA GAC GGC ATA CGA GAT GAT CTG GTG ACT GGA GTT CAG ACG TGT GCT CTT CCG ATC TGG TAA GGC TTT GGA GAA CCC A

HS-F11	HiSeq	CAA GCA GAA GAC GGC ATA CGA GAT TCA AGT GTG ACT GGA GTT CAG ACG TGT GCT CTT CCG ATC TGG TAA GGC TTT GGA GAA CCC A
5PGK probe	northern blotting	AAC TGG AGC CAA AGA GTA TTT TTC GTT TCT TTC ACC GTT TGG TCT ACC CAA GTG AGA AGC
oRP121 probe	northern blotting	AAT TCC CCC CCC CCC CCC CCC CA
SCR1 probe	northern blotting	GTC TAG CCG CGA GGA AGG
PGK-CGA12-F	cloning	CTAGA CGA CGA CGA CGA CGA CGA CGA CGA CGA CGA CGA CGA T
PGK-CGA12-R	cloning	CTAGA TCG TCG TCG TCG TCG TCG TCG TCG TCG TCG TCG TCG T
PGK-AAA12-F	cloning	CTAGA CGC AAA AAA AAA AAA AAA AAA AAA AAA AAA AAA AAA AAA T
PGK-AAA12-R	cloning	CTAGA TTT TTT TTT TTT TTT TTT TTT TTT TTT TTT TTT TTT GCG T
SKI7::Leu-2-F	knockout cassette	CGA GGA GGT GGT CTT CGA AAC TTA CAG TAC CAC CTG ACG TTA ACT GTG GGA ATA CTC AGG
SKI7::Leu-2-F-1	knockout cassette	GAT TGG AGG TAT AAA CCT AGA GAC CCT TCT ACA ATA CAC GTA CGA GGA GGT GGT CTT CGA
SKI7::Leu-2-R	knockout cassette	TAA GTA TGA ATG CCT AGT ATA ATT TCT TAG TTG TAG GAT TGA CTT AAA CTC CAT CAA ATG
SKI7::Leu-2-R-1	knockout cassette	CAA CTT ATT ACT ATT CAT TTT ATA TAT TAA ACA ATA AGT ATG AAT GCC TAG TAT AAT TTC
Rps3-F (BamHI)	cloning	ACT GAG TTC GGA TCC GTC GCT TTA ATC TCT AAG AA
Rps3-R (XhoI)	cloning	CAA ATC TGG CTC GAG CTA AGC TTC AAC TGG TTC AGC TTG AGC T
Rps3-UTR-5’:	cloning	GCT GAA CCA GTT GAA GCT TAG
Rps3-UTR-3’:	cloning	CCT AGC GGA TCT GCC GGA GAA AGA TGA ACT GCT ACT CAC T
Rps3-UTR-His-5’	cloning	AGT GAG TAG CAG TTC ATC TTT CTC CGG CAG ATC CGC TAG G

Rps3-UTR-His-3’	cloning	AAC TCA TTC ATA TCC GAG AAA TCG TCC TGT AAG CTG GAA TTC GAG CTC GTT TAA ACT GGA
Rps3-UTR-His-3’-2	cloning	TAC AGG CGG CGT ATA CAA GTG GTG AAA ACG ATA GCG AAC TCA TTC ATA TCC GAG AAA TCG
R116117A-F	site-directed mutagenesis	GTT GAA CGG TTT GGC TAT CGC TGC TGC TGC TTA CGG TGT CGT CAG
R116117A-R	site-directed mutagenesis	CTG ACG ACA CCG TAA GCA GCA GCA GCG ATA GCC AAA CCG TTC AAC
Rps2-F (BamHI)	cloning	ACTGAGTTCGGATCCATGTCTGCTCCAGAAGCTCA
Rps2-R (XhoI)	cloning	GCTAATGACCTCGAGCTCCACCTCCGGTTGAAAAG
Rps2-E120A-F	site-directed mutagenesis	GTATCAAGACCGCCAAGGCAGTTGCTGGTGCCATCAG
Rps2-E120A-R	site-directed mutagenesis	GTATCAAGACCGCCAAGGCAGTTGCTGGTGCCATCAG
ASC1-F(XbaI)	cloning	CAA TAT TTA CTC TAG ATG CAC CAT TCT ACG
ASC1-R(BamHI)	cloning	GAT CAA CTG GAT CCT TCA ATT GCA CAG TC
ASC1-R38D-K40E-F	site-directed mutagenesis	CCT ATT GTT GTC CGC TTC CGA CGA CGA GAC TTT GAT CTC CTG GAA G
ASC1-R38D-K40E-R	site-directed mutagenesis	CTT CCA GGA GAT CAA AGT CTC GTC GTC GGA AGC GGA CAA CAA TAG G
